# Membrane associated proteins of two *Trichomonas gallinae* clones vary with the virulence

**DOI:** 10.1371/journal.pone.0224032

**Published:** 2019-10-24

**Authors:** María del Carmen Martínez-Herrero, María Magdalena Garijo-Toledo, Fernando González, Ivana Bilic, Dieter Liebhart, Petra Ganas, Michael Hess, María Teresa Gómez-Muñoz

**Affiliations:** 1 Departamento de Producción y Sanidad Animal, Salud Pública Veterinaria y Ciencia y Tecnología de los Alimentos, Facultad de Veterinaria, Instituto de Ciencias Biomédicas, Universidad CEU Cardenal Herrera, Valencia, Spain; 2 GREFA—Grupo de Rehabilitación de la Fauna Autóctona y su Hábitat, Majadahonda, Madrid, Spain; 3 Clinic for Poultry and Fish Medicine, Department for Farm Animals and Veterinary Public Health, University of Veterinary Medicine Vienna, Vienna, Austria; 4 Departamento de Sanidad Animal, Facultad de Veterinaria, Universidad Complutense de Madrid, Madrid, Spain; Consejo Superior de Investigaciones Cientificas, SPAIN

## Abstract

Oropharyngeal avian trichomonosis is mainly caused by *Trichomonas gallinae*, a protozoan parasite that affects the upper digestive tract of birds. Lesions of the disease are characterized by severe inflammation which may result in fatality by starvation. Two genotypes of *T*. *gallinae* were found to be widely distributed in different bird species all over the world. Differences in the host distribution and association with lesions of both genotypes have been reported. However, so far no distinct virulence factors of this parasite have been described and studies might suffer from possible co-infections of different genotypes. Therefore, in this paper, we analyzed the virulence capacity of seven clones of the parasite, established by micromanipulation, representing the two most frequent genotypes. Clones of both genotypes caused the maximum score of virulence at day 3 post-inoculation in LMH cells, although significant higher cytopathogenic score was found in ITS-OBT-Tg-1 genotype clones at days 1 and 2, as compared to clones with ITS-OBT-Tg-2. By using one representative clone of each genotype, a comparative proteomic analysis of the membrane proteins enriched fraction has been carried out by a label free approach (Data available via ProteomeXchange: PXD013115). The analysis resulted in 302 proteins of varying abundance. In the clone with the highest initial virulence, proteins related to cell adhesion, such as an immuno-dominant variable surface antigen, a GP63-like protein, an armadillo/beta-catenin-like repeat protein were found more abundant. Additionally, Ras superfamily proteins and calmodulins were more abundant, which might be related to an increased activity in the cytoskeleton re-organization. On the contrary, in the clone with the lowest initial virulence, larger numbers of the identified proteins were related to the carbohydrate metabolism. The results of the present work deliver substantial differences between both clones that could be related to feeding processes and morphological changes, similarly to the closely related pathogen *Trichomonas vaginalis*.

## Introduction

*Trichomonas gallinae* is a protozoan that affects the upper digestive tract of domestic and wild birds, although columbiformes are considered the principal reservoir of the parasite. The main route of transmission of this flagellated microorganism among columbiformes is feeding chicks with crop milk or sharing food or water sources. Recently, *T*. *gallinae* has been recognized as the etiological agent of epidemic outbreaks of disease affecting wild finches that spread across Europe and North America [1].

Although *T*. *gallinae* was described as the etiological agent of avian trichomonosis for some time, the advancement of molecular epidemiology has elucidated the diversity of trichomonads present in the oropharynx of birds in the recent decades. Several genotypes of *T*. *gallinae* have been described in distinct studies, employing different nomenclature [2–6]. In addition, the occurrence of new or tentative species has been reported, some of them associated with particular avian hosts [2, 5, 7–10]. However, not all of them have been related to lesions and disease. Several factors can influence the development of pathognomonic lesions or clinical signs associated with avian trichomonosis. Among them, the age, previous contact with the parasite, diet and type of host have been reported [1, 6].

Two genotypes of *T*. *gallinae* are widely distributed among several groups of birds: genotypes A and C [2] which correspond with genotypic groups Internal Transcribed Spacer-Oropharyngeal Bird Trichomonad-*T*.*gallinae* 1 (ITS-OBT-Tg-1) and Internal Transcribed Spacer-Oropharyngeal Bird Trichomonad-*T*.*gallinae* 2 (ITS-OBT-Tg-2) [6]. Although both genotypes have been described in clinical cases, ITS-OBT-Tg-1 appears to be more frequently associated with disease [3, 6, 11–13]. However, the virulence of the distinct genotypic groups has been only partially investigated [14].

So far only two studies have been carried out on the proteomics of *T*. *gallinae* [15, 16]. One of them identified cysteine peptidases related to the pathogenic effect of the parasite in cell cultures [15], while the other predicts glyceraldehyde-3-phosphate hydrogenase (GAPDH) as a promising drug target [16]. In UniProt database there are only 71 annotations for *T*. *gallinae*, corresponding to 9 proteins. On the contrary, several proteomic studies have been performed with the closely related protozoan *Trichomonas vaginalis*, the etiological agent of the human venereal disease called trichomonosis and more than 50000 proteins have been annotated in UniProt. Some studies have focused on the composition of surface proteins of *T*. *vaginalis*, since they are involved in several mechanisms of inter-cell communication, such as nutrient acquisition, adhesion to epithelial cells and different virulence mechanisms [17–23]. It is known that several surface protein families are present in *T*. *vaginalis*: *BspA*-like proteins, surface proteases, variable surface proteins and lectins, together with proteins harboring dual functions in the parasitic cell. *BspA*-like proteins act by binding to epithelial cells in many organisms, including Bacteria, Archea and Eukaryota [17]. Surface proteases participate in inflammation processes, neoplastic mechanisms and cell adhesion, and include cysteine proteases, serine proteases and metalloproteinases such as GP63-like proteins [17, 19, 20–22]. Proteins with dual functionality are called moonlighting proteins, since they are present in different compartments of the cell, including the plasmatic membrane. Among them, GAPDH family and adhesins, a group that englobes the malic enzyme, have been described in *T*. *vaginalis* [17, 21].

The membrane system of the cells is composed of the cell outer membrane and associated proteins, including proteins of the membrane associated organelles. Extensive vesicular traffic occurs connecting the organelles one to each other and also to the outside of the cells and many signaling pathways take place there. Although some membrane proteins are known to be present in both, *T*. *vaginalis* and *T*. *gallinae*, it is reasonable to expect that many others are specific and not easily identifiable by comparison, due to the difference in host (man and birds).

In this paper, we have chosen seven clones of the two main genotypic groups of *T*. *gallinae*, and analyzed the virulence by *in vitro* cell culture-based assay. According to their initial virulence, two isolates were further selected for a comparative proteomic analysis. The ability of *T*. *gallinae* to change its shape and adhere to epithelial cells, together with excretion of cysteine peptidases, are essential characteristics for virulence [1, 15]. Thus, the knowledge of the protein composition of the membrane is crucial to elucidate those processes. Therefore, the aim of the present work was to analyze differences in the enriched membrane proteins fraction after subcellular fractionation of the trophozoites.

## Material and methods

### Isolates, culture and preservation

Isolates of *T*. *gallinae* were obtained in the context of routine diagnosis of avian trichomonosis in the wildlife recovery center of GREFA (Madrid, Spain). Veterinary supervision and care of wild animals, as well as samples for disease diagnosis, are routine procedures for all the animals at admission. Bird samples were collected by veterinarians by gentle rubbing sterile cotton swabs in the oropharynx of birds and subsequently cultured in 5 ml of fresh TYM (Trypticase Yeast Maltose) medium pH 6.5 at 37°C containing 10% inactivated fetal bovine serum (FBS) (Sigma-Aldrich, St. Louis, Missouri, USA), antibiotics (36 mg/l each of ticarcillin, vancomycin and ceftiofur) and 10,000 IU/ml nystatin (Sigma, St. Louis, Missouri, USA). The tubes were monitored daily using an inverted microscope for the presence of viable trichomonads, for a maximum period of 15 days. Aliquots of 1 ml of each culture were kept for DNA isolation of the original isolate.

Passages were performed every 48 hours using 100 μl of sample, until bacterial or fungal contamination was controlled, usually after 3–6 passages. The presence of bacteria and fungi was examined using Nutrient agar (Scharlab, S.L., Barcelona, Spain) and Sabouraud agar (bioMérieux, Madrid, Spain) by plating 100 μl of culture on each agar plate. Nutrient agars were incubated at 37°C for 24 hours, while Sabouraud agars were maintained preserved from light at 22°C for 15 days.

Preservation of the isolates was done using 5% of dimethyl sulfoxide (DMSO, Sigma-Aldrich, St. Louis, Missouri, USA) in TYM medium. The cryovials were placed in a freezing container that provided a slow cooling rate (-1°C/min) in a -80°C freezer. For long term storage (>12 months) samples were transferred into a -150°C freezer.

### Establishment of clonal cultures

The isolates used in the present work were selected based on their origin: three isolates were obtained from animals with macroscopic lesions characteristic for avian trichomonosis and four isolates were obtained from animals without macroscopic lesions (Table 1).

**Table 1:** MLST of selected clonal cultures.

ITS Type (2, 6)	SSU-Type (4)	Fe.hydrogenase Type (13)	Clone^1^	Gross Lesions^2^	Host
A (2) ITS-OBT-Tg-1 (6)	KM095107	A1 (JF681136)	R193-13 C3	Yes	*Falco tinnunculus*
			P95-13 C3	No	*Streptopelia decaocto*
			P349-12 C11	Yes	*Columba palumbus*
	18S-VI (FN433485)		R17-12 C1		*Bubo bubo*
C (2) ITS-OBT-Tg-2 (6)	18S-II (EU215373)	C4 (KC529662)	R44-12 C5	No	*Accipiter gentilis*
	18S-II (EU215374)	C2.1 KP900032	R24-12 C10		*Falco tinnunculus*
	18S-II (EU215373)	C4 (KC529662)	P178-13 C7		*Columba palumbus*

MLST of clonal cultures selected for virulence assays in LMH cells. References are included in parenthesis.

^1^ Clone Code, the first letter refers to bird species prey (P) or raptor (R). The first two or three digits refer to the laboratory internal code and the last two digits to the sampling year. The letter C refers to the number of clone obtained from each isolate.

^2^ Gross Lesions in the oropharyngeal cavity.

Each isolate was thawed in LMH culture medium consisting of medium 199 (M199) supplemented with Earle´s salts, 100 mg/ml L-glutamine, 25mM HEPES, L-amino acids (Gibco^™^, Thermo Fisher Scientific, Vienna, Austria), 0.22% of sterilized rice starch (Carl-Roth, Karlsruhe, Germany) and 15% of heat inactivated fetal bovine serum (Sigma, EEUU). *Escherichia coli* DH5α-T1 incubated in a horizontal rotating incubator at 37°C in the supplemented medium for 24 hours was added to the cultures in a proportion of 1/20 (v/v) (LMH culture medium), before inoculation of the parasites. Prior to the establishment of the clonal culture by a previously described micromanipulation procedure [24], the original isolates were cultivated for a short period of time (maximum 7 passages). Passages of the cultures were done every 48–72 hours.

One day before the micromanipulation procedure, 0.5 ml of each *Trichomonas* isolate culture was transferred into 9 ml of LMH culture medium supplemented with *E*. *coli*. At 24 hours post-inoculation, 20 μl of these cultures were carefully placed on glass slides and diluted with pure M199 in order to have a clear suspension of trophozoites. The selection of single trichomonad cells was done under an inverted microscope (Diaphot 300, Nikon, Austria) by a micromanipulator (Narishige, Japan). Single active trophozoites were retained by negative pressure with a micropipette. Each single trophozoite was deposited in a drop of M199 to visualize the correct selection of the cell and then transferred to a micro tube with 0.5 ml of LMH culture medium supplemented with *E*. *coli*. The tubes were incubated at 37°C and examined every 48 hours for trophozoite growth. Positive clonal cultures were transferred into 9 ml of LMH culture medium supplemented with *E*. *coli* for successive culturing and cryo-preservation until use.

Clones of the genotypic group ITS-OBT-Tg-1, obtained from animals with lesions, including R17-12 C1 from a Eurasian eagle-owl (*Bubo bubo*), R193-13 C3 from a common kestrel (*Falco tinnunculus*) and P349-12 C11 from a common wood pigeon (*Columba palumbus*). Clones of the genotypic group ITS-OBT-Tg-2, obtained from animals without lesions, including P178-13 C7 from a common wood pigeon (*Columba palumbus*), R24-12 C10 from a common kestrel (*Falco tinnunculus*) and R44-12 C5 from a northern goshawk (*Accipiter gentilis*). One isolate (P95-13 C3) that belonged to genotype ITS-OBT-Tg-1 was obtained from a bird without clinical signs. The information about clones, including host species of origin, presence of macroscopic lesions and ITS genotypes is shown in Table 1. From each isolate, between 6 and 25 clones were successfully established. One clone from each isolate was selected for the virulence assays.

#### Axenization procedure

Since clonal *T*. *gallinae* trophozoites were cultured in the presence of *E*. *coli*, penicillin, streptomycin and meropenem (Sigma-Aldrich, St. Louis, Missouri, USA) were added for the axenization procedure according to Amin et al. [24]. Tryptose-soy-agar (TSA) and tryptose-phosphate-broth with 0.2% of agar (TPB, Gibco^™^, Thermo Fisher Scientific, California, USA) were used to monitor bacterial and fungal contamination (Scharlab, S.L., Barcelona, Spain). TSA plates and TPB tubes were incubated 24 h at 37°C before examination. Once the trophozoites were growing axenically, they were transferred to Hollander Fluid medium at pH 7.2 (HF medium) [24].

### DNA isolation and genetic characterization of the isolates and clonal cultures

DNA was extracted from 1 ml of the initial parasite culture or at passage level <3. From clonal cultures, 1 ml was also used for DNA extraction after the axenization process was completed. DNeasy blood and tissue extraction kit (QIAGEN, Valencia, California, USA) was employed following the manufacturer´s instructions and DNA was stored at -20°C until use.

#### ITS1/5.8S rRNA/ITS2 (ITS) PCR

The protocol of Felleisen [25] was applied for amplification and sequencing of the ITS1/5.8S rRNA/ITS2 region using primers TFR1 (5´-TGCTTCAGCTCAGCGGGTCTTCC-3’) and TFR2 (5’-CGGTAGGTGAACCTGCCGTTGG-3’). PCR reaction was carried out in 50 μl with 5 μl of 10x PCR buffer, 1.5 mM of MgCl_2_, 2 mM of dNTP, 2 μM of each primer, 2.5 IU of Taq polymerase (MP, Thomas Scientific, Swedesboro, New Jersey, USA) and 5 μl of genomic DNA. PCR protocol was as follows: initial denaturation step at 95°C for 9 min followed by 40 cycles of denaturation for 30 s at 94°C, annealing at 66°C for 30 s, extension at 72°C for 30 s and a final extension step at 72°C for 15 min. Positive and negative controls were included in each PCR reaction set.

#### Small subunit of ribosomal RNA (SSUrRNA) PCR

Amplification of the SSUrRNA was carried out using the protocol of Ganas et al. [26] and the primers HM long f (5′-AGGAAGCACACTATGGTCATAG-3′) and HM long r (5′-CGTTACCTTGTTACGACTTCTCCTT-3′). The PCR was performed in 50 μl composed of 25 μl of HotStarTaq Master Mix kit polymerase (QIAGEN, Hilden, Germany), 16 μl of PCR water (QIAGEN, Hilden, Germany), 2 μl of each primer at 10 μM and 5 μl of genomic DNA. The temperature profile was as follows: 15 min at 95°C for initial denaturation, 40 cycles of 30 s at 94°C, 1 min at 55°C, 2 min at 72°C and a final extension step of 10 min at 72°C. Positive and negative controls were included in each PCR reaction set.

#### Fe-hydrogenase gene (FeHyd) PCR

For this genetic marker the same PCR reaction mix as for the SSUrRNA was prepared according to Lawson et al. [12] with the following primers: TrichhydFOR (5´-GTTTGGGATGGCCTCAGAAT-3´) and TrichhydREV (5´-AGCCGAAGATGTTGTCGAAT-3´). The thermal cycler was programmed for: 15 min at 95°C for initial denaturation, 40 cycles of 30 s at 94°C, 1 min at 58°C, 2 min at 72°C and lastly, 10 min at 72°C. Positive and negative controls were included in each PCR reaction set.

*Sequencing*. Amplicons were purified with a MiniElute PCR purification kit (QIAGEN, Valencia, California, USA) and submitted for Sanger sequencing to Sistemas Genómicos S.A., Valencia, Spain. An automatic sequencer (3730XL DNA analyzer, Applied Biosystems, Foster City, California, USA) was employed using the ABI PRISM BigDye© Terminator Cycle sequencing kit (Applied Biosystems, Foster City, California, USA). Sequencing was carried out in both directions of DNA and the resultant chromatograms were assembled and manually checked using Lasergene SeqMan software version 7.0.0 (DNASTAR, Madison, Wisconsin, USA). Alignment was done using the BLAST online resource of the NCBI.

### Virulence assays of *T*. *gallinae* clonal cultures in LMH cells

#### LMH cell culture

The chicken hepatocellular carcinoma cell line (LMH) was used for virulence assays, since it provided a suitable system for the *in vitro* pathogenicity testing of *T*. *gallinae* [14]. LMH cells were cultured in RPMI-1640 supplemented with 10% FBS, 40,000 IU/ml penicillin and 40 mg/ml streptomycin (Gibco^™^, Thermo Fisher Scientific, California, USA). Cells were incubated in 5% CO_2_ and 85–90% humidity at 37°C.

Ten ml of medium with an initial inoculum of 1.4 x 10^6^ cells were cultured in 25 cm^2^ filtered cap flasks (Nunc^™^, Thermo Fisher Scientific, California, USA). The cultures were split every 3–4 days by adding 2 ml of a trypsin solution (0.05% of trypsin in 0.53 mM EDTA, Thermo Fisher Scientific, California, USA) for 3–5 min followed by washing of the cells in 50 ml of sterile PBS (pH 7.2). After centrifugation at 1,300 rpm for 5 minutes the cells were suspended in 10 ml of fresh medium. To assess cell viability, trypan blue exclusion was used (Sigma-Aldrich, St. Louis, Missouri, USA).

#### Inoculation of LMH cells

Prior to the inoculation with trichomonads, LMH cell cultures were washed with sterile PBS and 6.5 ml of pre-warmed M199 supplemented with 10% FBS, 10% TPB and penicillin-streptomycin solution. LMH cells were used for inoculation of trophozoites when 95% confluence was obtained (monolayer stage).

An inoculum of 10,000 axenized viable trophozoites was used in each flask of LMH cell cultures in monolayer. Three replicates were performed for each clone together with flasks with solely trophozoites as positive controls and flasks with solely LMH cells as negative controls. The cultures were incubated for five days and the number of trophozoites was determined every 24 hours.

#### Determination of cytopathogenic effect

The cultures were monitored by an inverted microscope for five days to assess the cytopathogenic effect. In parallel, the parasites were counted by trypan blue exclusion every 24 hours. Cytopathogenic effects (CPE) were scored according to Amin et al. [14]: CPE1: ≤25% of monolayer destruction, CPE2: 25–50% of monolayer destruction, CPE3: 50–75% of monolayer destruction and CPE4: >75% of monolayer destruction.

#### Statistical analysis

Wilcoxon signed rank test was employed for statistical significance in the growth of trophozoites with or without LMH cells. Categorical data for CPE scores grouped by ITS genotype were analyzed using the Chi square test of association (*p*-value < 0.01). All analyses were performed with the IBM SPSS Statistics software version 22 (IBM Corp., Armonk, New York, USA).

### Subcellular fractionation and protein extraction and quantification

Based on the virulence assays, two of the clonal cultures of *T*. *gallinae* were selected for the proteomic analysis: at 24h p.i. clone R17-12 C1 showed the highest CPE score (4) whereas P178-13 C7 caused the lowest CPE score (1).

#### *T*. *gallinae* mass culture and harvest

Three biological replicates were performed with each clone. The clones were thawed in LMH culture medium supplemented with *E*. *coli* at 37°C. The parasites were progressively axenized as previously described and adapted to TYM medium. Mass cultures were generated by the inoculation of 1 x 10^5^ axenic trophozoites in 40 ml of TYM medium on 20 Falcon tubes of 50 ml and harvested at the end of the log phase (48 hours). In this case, trophozoites grew in TYM medium without antibiotics and antifungals. Clone R17-12 C1 was at passage 9 and clone P178-13 C7 at passage 11. Cell viability was assessed at 24 and 48 hours by trypan blue exclusion. Between 1.1 x 10^8^ and 1.7 x 10^7^ trophozoites were harvested from each Falcon tube by centrifugation at 2,000 rpm for 5 min and then washed 3 times with sterile PBS (pH 7.2) at 4°C to remove growth medium. The obtained cell-pellets were used for subsequent subcellular fractionation.

#### Subcellular fractionation

The commercial subcellular fractionation kit ProteoExtract^™^ Subcellular Proteome Extraction kit (Calbiochem^™^, Merck KGaA, Darmstadt, Germany) was employed for this purpose. Manufacturer instructions were followed to avoid protein degradation. Briefly, cells were suspended with protease inhibitor cocktails and a series of extraction buffers that dissolved different proteome fractions were applied. Firstly, soluble proteins corresponding to the cytosolic fraction were extracted. Then, the fraction containing membrane and membrane-bound organelles proteins was obtained. Thirdly, nuclear proteins and the cytoskeletal fraction were recovered. During the procedure the samples were kept on ice to avoid protein degradation. Incubation was done in mechanical agitators to assess the correct distribution and solubilization of the extraction buffers. The obtained fractions were kept at -80°C for further analysis. Fraction two (enriched membrane proteins fraction) was used for this study.

#### Protein precipitation and quantification

The enriched membrane proteins fraction was gradually thawed from -80°C to 4°C and kept on ice for acetone precipitation. Six volumes of acetone at -20°C were added to one volume of sample. The mix was kept at -20°C for 4 hours and then centrifuged at 8,000 rpm for 10 minutes. Acetone was carefully poured from the tube and the pellet was kept at -80°C. For solubilization, samples were suspended in 100 μl of 8M urea. Protein quantification was done by the RC DC^™^ Protein Assay (Bio-Rad Laboratories, Hercules, California, USA). The same amount of protein was used for each replicate of the clones before performing further analysis.

#### SDS-PAGE preliminary analysis

A preliminary analysis by a 12% SDS-PAGE gel using 10 μg/lane of each sample from the enriched membrane proteins fraction of the two selected clones were loaded to compare the protein pattern of the clones. The same amount of protein was loaded from each replicate as a quality control for the protein quantification. Precision Plus Protein^^™^^ Standards Dual Color de (Biorad, Alcobendas, Spain) were used as molecular weight markers.

#### Digestion and desalting of the peptides

Thirty mg of sample from each replicate were reduced with 10 mM of dithiothreitol (DTT) at 37°C for 30 minutes. Proteins were alkylated with 55 mM of iodocetamide in the dark for 20 min. Protein digestion was carried out with 1/30 (w/w) of trypsin (recombinant trypsin sequencing grade, Roche) and left overnight at 37°C [27]. Digested peptides were desalted and concentrated by reverse chromatography (C18 OMIX pipette tips, Agilent Technologies, Germany). Peptides were eluted with 50% acetonitrile (ACN) and 0.1% of trifluoroacetic acid. Finally, the samples were dried in a speed-vac and resuspended in 0.1% of formic acid before de Nano LC-MS/MS analysis. Samples were stored at -20°C for further procedures.

#### Label free experimental design

**Fig 1:**
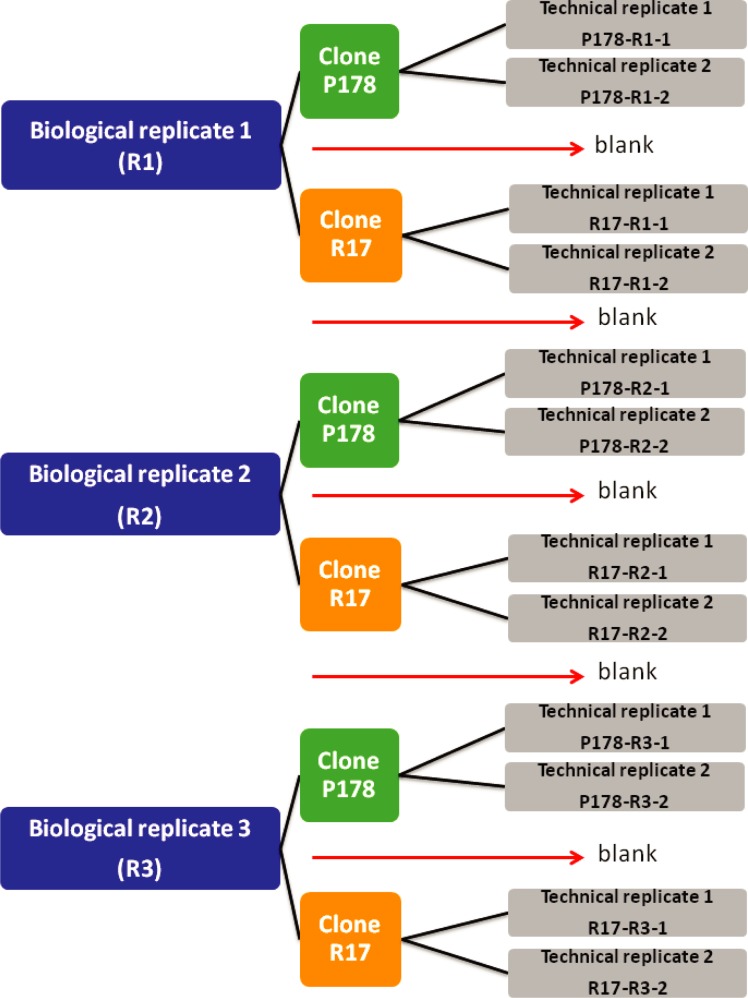
Experimental design for comparative label free proteomic analysis. Experimental design for label free proteomic analysis comparing identified proteins of the enriched membrane protein fraction of clones R17-12 C1 and P178-13 C7.

Three biological replicates were used from each clone. A total of two technical replicates of each biological replicate were analyzed, except for one technical replicate of clone R17-12 C1 (R17-R1-2) which had an insufficient amount of protein. From clone R17-12 C1 samples R17-R1-1, R17-R2-1, R17-R2-2, R17-R3-1 and R17-R3-2 were employed, while from clone P178-13 C7 samples P178-R1-1, P178-R1-2, P178-R2-1, P178-R2-2, P178-R3-1 and P178-R3-2 were used (Fig 1).

#### LC-MS/MS analysis (LTQ-Orbitrap-Velos-Pro)

The desalted peptides samples were analyzed by RP-LC-MS/MS in an Easy-nLC II system coupled to an ion trap LTQ-Orbitrap-Velos-Pro mass spectrometer (Thermo Fisher Scientific, California, USA). The peptides were concentrated (on-line) by reverse phase chromatography using a 0.1 mm × 20 mm C18 RP precolumn (Thermo Scientific, California, USA), and then separated using a 0.075 mm x 250 mm C18 RP column (Thermo Scientific, California, USA) operating at 0.3 μl/min. Peptides were eluted using a 180-min gradient from 5 to 40% solvent B (Solvent A: 0.1% formic acid in water, solvent B: 0.1% formic acid and 80% ACN in water). For peptide electrospray ionization (ESI) a nanoelectrospray source and 30 mm capillary emitters were employed (Nano-bore emitters stainless steel ID 30 μm, Proxeon, Thermo Fisher Scientific, California, USA).

The mass spectrometer was run in data-dependent positive ionization for automatically detection from MS to MS/MS spectra (LTQ-Orbitrap-Velos-Pro). Peptides were detected with a 30,000 resolution in full scan MS mode with 400–1,600 atomic mass units (amu). A maximum of 15 dependent MS/MS scans were selected according to their intensity. An isolation width of ± 2 mass-to-charge ratio units (m/z), normalized collision energy of 35% and dynamic exclusion were applied for periods of 30 seconds.

The mass spectrometry proteomics data have been deposited to the ProteomeXchange Consortium via the PRIDE [28] partner repository with the dataset identifier PXD013115 and 10.6019/PXD013115.

### Peptide identification and relative quantification

#### Peptide identification

Peptide identification from raw data was carried out using licensed version of search engine MASCOT 2.3.0 thorough Proteome Discoverer^™^ 1.4 (Thermo Fisher Scientific, California, USA). The non-redundant NCBI protein database with taxonomic restriction to family trichomonadidae (103,432 protein sequences) was selected for further analysis, since no specific database of *T*. *gallinae* was available and *T*. *vaginalis* is a phylogenetically closely-related organism with a broad database available. The search was optimized with the following criteria: 20 ppm of tolerance for precursor ions and 0.8 Da for MS/MS fragment ions, tryptic cleavage after arginine and lysine, up to two missed cleavage sites allowed, optional modification of methionine oxidation and fixed modification of carbamidomethylation of cysteine. Search against decoy database (integrated decoy approach) was used to False Discovery Rate (FDR) estimation and MASCOT percolator filter were applied to MASCOT results. Acceptance criteria for protein identification were FDR < 1% and at least two peptides identified with a confidence interval (CI) > 95% (*p*-value < 0.01).

#### Protein relative quantification

Progenesis QI software version 4.1 (Nonlinear Dynamics, Waters company, Newcastle upon Tyne, UK) was employed for the relative quantification of the identified proteins. This program detects and quantifies all the peptide ions produced in the LC-MS/MS and analyses protein expression. The abundance of a specific protein was calculated as the average normalized abundance of its unique peptides in each run, following the recommendations of the Progenesis users guide. Differential expressed proteins were considered those with a fold change ≥ 2 for their average value of normalized intensity ratio.

Predicted protein functions were assigned using the UniProtKB database which includes Swiss-Prot (551,987 protein sequences) and TrEMBL (66,905,753 proteins) (UniProt, 2016). BLAST analysis was performed and Gene Ontology (GO) information entries were used for the inference of biological functions (February 2019). The free software Cytoscape version 3.5.1. was used to represent the association of the identified proteins with their specific functions [29].

## Results

### Isolates and clonal cultures

Seven isolates obtained from the oropharynx of different species were cloned for further investigations of the virulence and subsequent proteomic experiments. Three of them were obtained from birds with lesions and one form a bird without lesions that belonged to genotypic group ITS-OBT-Tg-1. Three other isolates were obtained from animals without lesions and belonged to genotypic group ITS-OBT-Tg-2. Results of the Multilocus Sequence Typing (MLST) analysis of the clones indicated that sequences were identical to other sequences found in other studies, with the exception of 1 single nucleotide polymorphism (SNP) in the ITS region of clone P178-13 C7 (Table 1).

### Virulence assays and growth curves

#### Virulence assays in LMH cells

**Fig 2:**
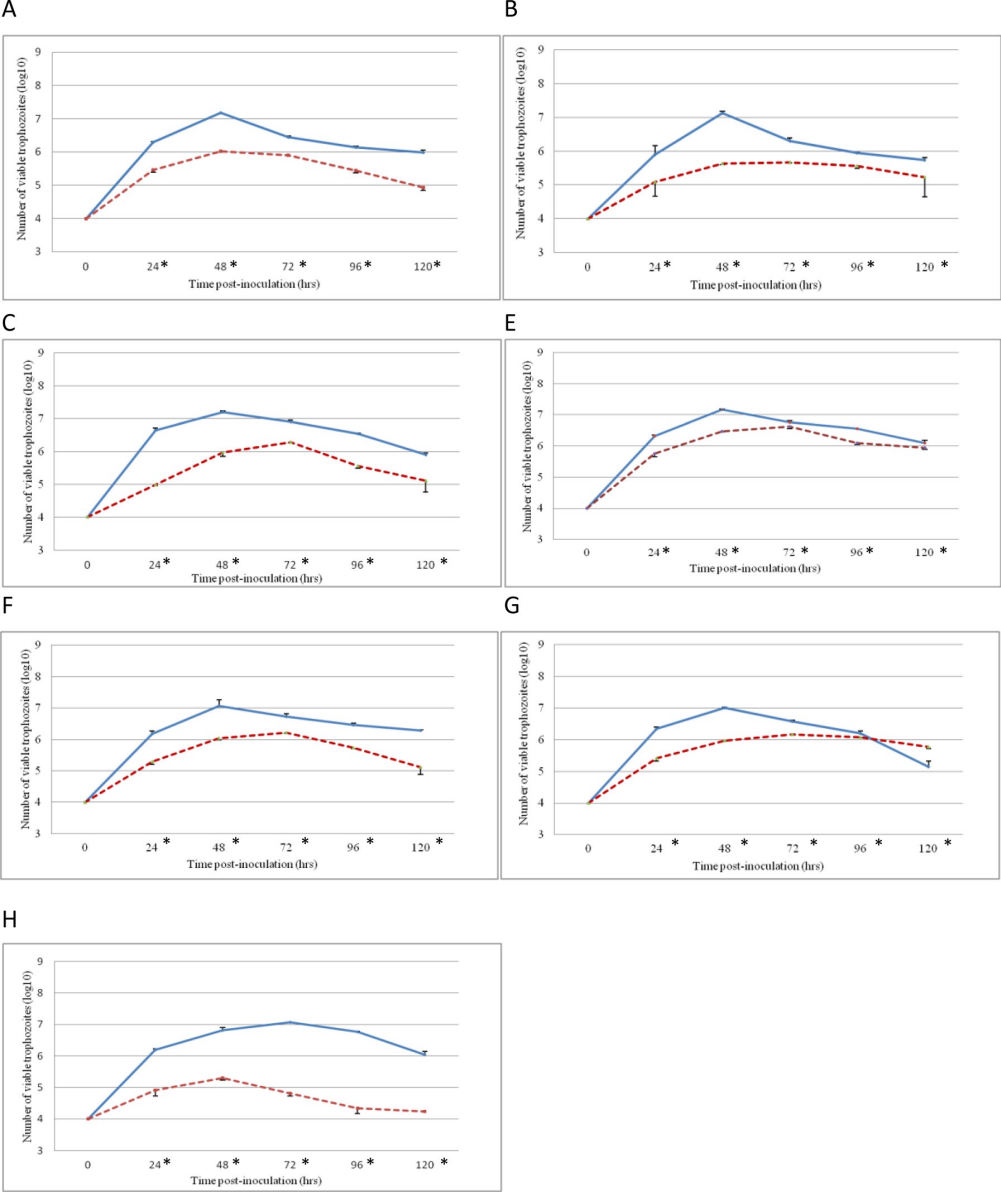
Growth curves of clones in LMH cells. Growth curves of *T*. *gallinae* trophozoites from clonal cultures on LMH cells. Error bars indicate standard deviation. Control flasks containing only trophozoites without LMH cells are indicated in discontinuous lines. A) isolate P349-12 C11, B) isolate P95-13 C3, C) isolate R17-12 C3, D) isolate R193-13 C3, E) isolate P178-13 C7, F) R24-12 C10, G) isolate R44-12 C5. (*) Significant differences (*p-* value<0.05) between each clone and its control were observed in all the clones.

For the majority of clonal cultures, trophozoites reached the maximum growth at 48 hours post-inoculum (p.i.) in the presence of Leghorn Male Hepatoma (LMH) cell line, with the exception of the clone R44/12 C5 that reached the maximum growth at 72 hours p.i. (Fig 2). After reaching the maximum growth, the number of viable trophozoites started to decrease. The average maximum number of viable trophozoites in the presence of LMH cells ranged between 1.3 x 10^7^ and 1.6 x 10^7^ parasites per flask (7 ml) for genotypic group ITS-OBT-Tg-1 clones, and between 1.0 x 10^7^ and 1.2 x 10^7^ trophozoites per flask for genotypic group ITS-OBT-Tg-2 clones (Fig 2). Trichomonads cultivated without LMH cells reached growth peaks at 48 or 72 hours p.i. (Fig 2 and S1 Data). However, the amount of trophozoites per flask was significantly lower than in the presence of LMH cells (*p*-value < 0.05).

Clones P349-12 C11 and R17-12 C1, of genotypic group ITS-OBT-Tg-1, produced the maximum cytopathogenic effect (CPE) score (4) as early as 24 hours p.i. (Table 2). The other two clones of the same genotypic group, P95-13 C3 and R193-13 C3, were not as virulent 24 hours p.i. and displayed CPE scores 3 and 2, respectively (Table 2). In contrast to the above mentioned clones of ITS-OBT-Tg-1, at 24 hours p.i. all three clones of genotypic group ITS-OBT-Tg-2 displayed low virulence with CPEs scores 1 and 2 (Table 2). At 48 hours, all clones of genotypic group ITS-OBT-Tg-1 reached the maximum score (4) whereas for clones of genotypic group ITS-OBT-Tg-2 the CPE score varied from 2 to 4 (Table 2). There were significant statistical differences on the average CPE score between genotypic groups ITS-OBT-Tg-1 and ITS-OBT-Tg-2 on day 1 and 2 (*p*-value < 0.01, Chi-square Test). However, from the day 3 p.i., all clones showed the maximum CPE (score 4) (Table 2). Two clones were selected for further studies, clone R17-12 C1, with the highest initial virulence, and clone P178-13 C7, with the lowest initial virulence.

**Table 2:** Cytopathogenic effect score on LHM cells.

Genotype		CPE score				
ITS (2, 6)	Clone (*In vitro* passage)^$^	Days post-infection				
		1#	2#	3	4	5
A (2)ITS-OBT-Tg-1 (6)	R193-13 C3 * (1 / 27)	2	4	4	4	4
	P95-13 C3 (2 / 27)	3	4	4	4	4
	P349-12 C11 * (2 / 12)	4	4	4	4	4
	R17-12 C1 * (0 / 25)	4	4	4	4	4
C (2)ITS-OBT-Tg-2 (6)	R44-12 C5 (3 / 14)	2	2	4	4	4
	R24-12 C10 (7 / 19)	1	4	4	4	4
	P178-13 C7 (1 / 27)	1	3	4	4	4

The score of cytopathogenic effect (CPE) detected on LMH cells infected with clonal *T*. *gallinae* cultures. Scores from 1 (low CPE) to 4 (maximum CPE) were applied. References are included in parenthesis.

*: indicates clonal cultures derived from isolates of birds with gross lesions.

^**$**^::*In vitro* passage of the isolate before cloning and after cloning

^#^: statistical significant differences between both genotypes (Chi-square test, *p*-value < 0.01).

#### Growth curves in TYM medium

Growth of trophozoites from the tow selected clones in TYM medium reached the maximum value at 72 hours p.i. Although a lower growth was observed in clone R1-12 C1, no significant differences were observed between clones at 24, 48 and 72 hours p.i. (S1 Fig and S1 Data).

### Protein identification

#### SDS-PAGE preliminary analysis

The 1D-SDS-PAGE gels showed minor differences in the protein profile between the two selected clones, consequently, a label-free method of comparison and identification was carried out (S2 Fig).

#### LC-MS/MS analysis

Clones R17-12 C1 and P178-13 C7 were selected according to their initial virulence on cell culture (24h and 72 h to reach the maximum CPE score, respectively) for the comparative analysis of the enriched membrane proteins fraction composition. The mean percentage of matched MS/MS spectra was closed to 2%. Similar chromatogram profiles were obtained for each biological and technical replicate of the same clonal culture, but substantial differences were observed between the peak profiles of the selected clones R17-12 C1 and P178-13 C7 (S3 Fig) (ProteomeXchange: PXD013115).

#### Quantification of relative protein abundance and predicted functions

Proteins identified in every technical and biological replicate from the two clonal cultures, containing more than 2 peptides and a *p*-value < 0.01 were quantified. Those with ≥ 2 fold change difference in abundance between the two clones were considered different in the analysis and those with ≥ 3 fold change difference in abundance were further discussed. In total, of the 750 identified proteins, 302 were found with ≥ 3 fold different abundance, of which 132 were more abundant in clone R17-12 C1 and 170 in clone P178-13 C7.

**Fig 3:**
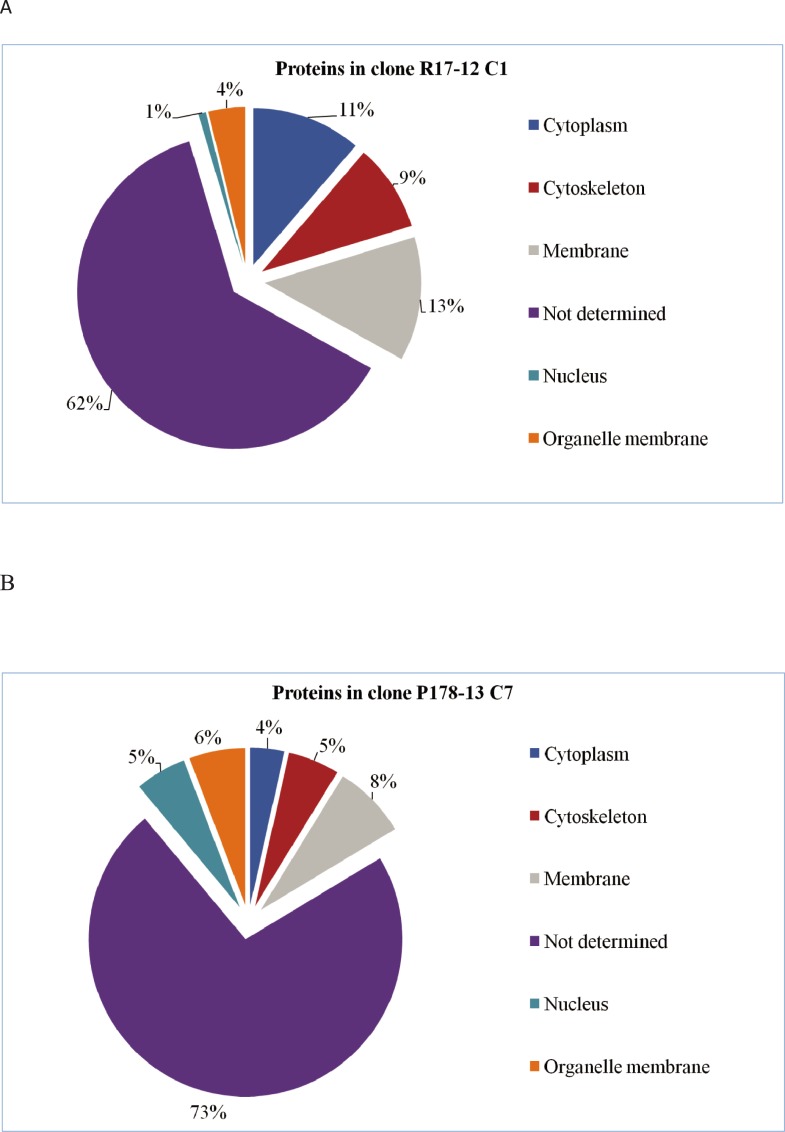
Identified proteins according to the subcellular location. Percentage of relatively quantified proteins more abundant A) in clone R17-12 C1 and B) in clone P178-13 C7 when compared to each other, classified according to their subcellular location.

For the majority of proteins, no information about their subcellular location was available (62% in clone R17-12 C1 and 73% in clone P178-13 C7) (Fig 3). Among the identified proteins more abundant in the ITS-OBT-Tg-1 clone (highest initial virulence score), a higher percentage of cell membrane, cytoskeleton and cytoplasm proteins were detected when compared with ITS-OBT-Tg-2 clone (lowest initial virulence score) (13%, 9% and 11% respectively in clone R17-12 C1 *vs*. 8%, 5% and 4% in clone P178-13 C7). On the contrary, ITS-OBT-Tg-2 showed a higher percentage of organelle membrane proteins and nuclear proteins (4% and 1% in clone R17-12 C1 *vs*. 6% and 5% respectively in clone P178-13 C7).

**Fig 4:**
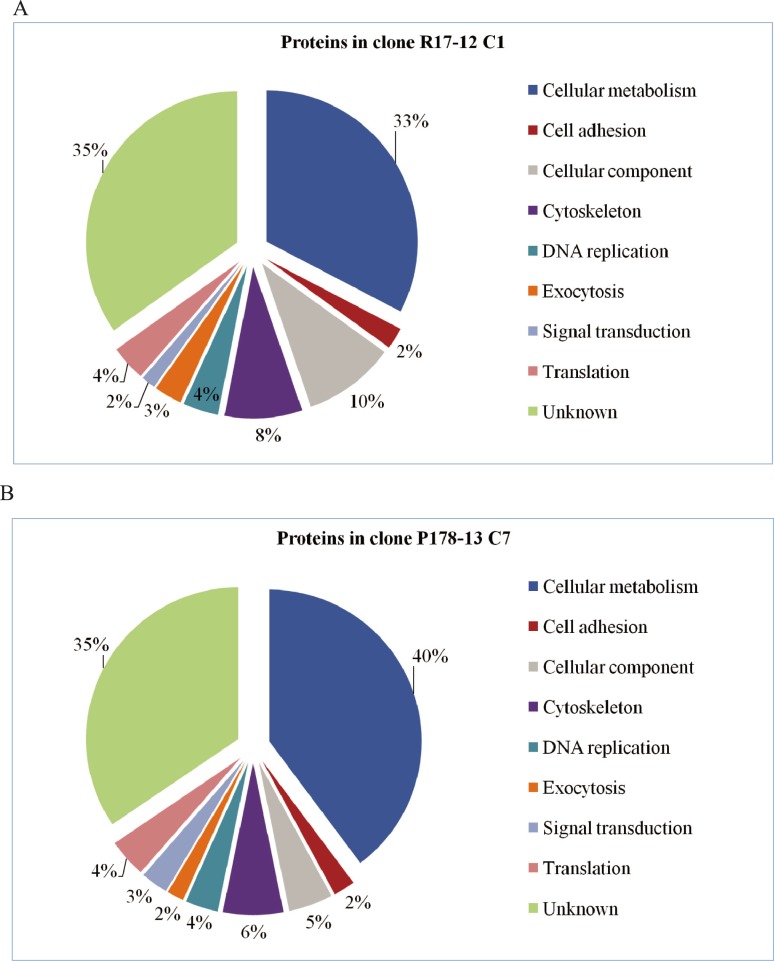
Identified proteins according to their predicted function. Percentage of relatively quantified proteins more abundant A) in clone R17-12 C1 and B) in clone P178-13 C7 when compared to each other, classified according to their predicted function category.

The group of proteins involved in the cellular metabolism showed the highest abundance, with 33% and 40% of the identified proteins in clones of genotypic groups ITS-OBT-Tg-1 and ITS-OBT-Tg-2, respectively (Fig 4). A large amount of the proteins, being present in both clones, for which no function could be predicted, was retrieved (35%). The percentages of proteins that differed in abundance between both clones and which participate in cell adhesion, DNA replication, exocytosis, signal transduction or translation were similar (2%, 4%, 3/2%, 2/3% and 4%, respectively). Among the identified proteins more abundant in the ITS-OBT-Tg-1 clone, a larger percentage from cellular components and cytoskeleton were identified (10% and 8% in clone R17-12 C1 *vs*. 5% and 6% in clone P178-13 C7).

#### Identified proteins

The largest group of identified proteins corresponded to hypothetical proteins as inferred from the genome of *T*. *vaginalis* isolate G3 [30], 42.4% in clone R17-12 C1 and 44.1% in clone P178-13 C7, respectively (S1 and S2 Tables). Although the majority could not be linked to any category or predicted function, some of the proteins were connected to cytoskeleton, protein metabolism, vacuoles, mitochondria, Golgi apparatus, or assembly of ribosomes. Identified proteins based on peptide sequences that differed in abundance between clones were grouped according to their category.

**Fig 5:**
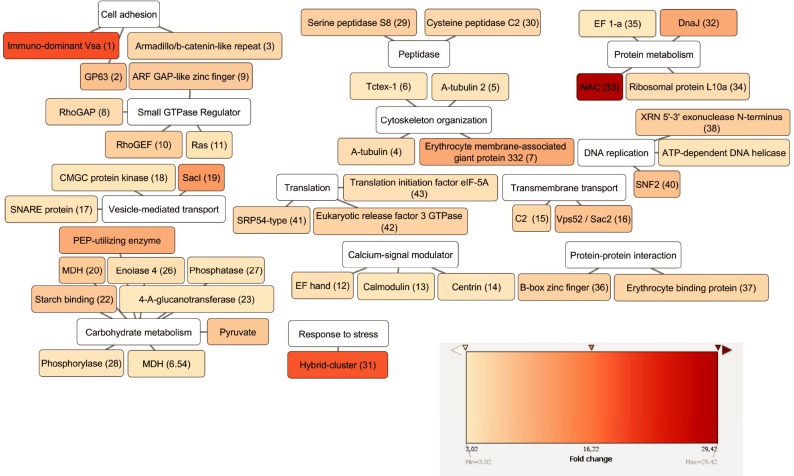
Proteins more abundant in R17. Proteins more abundant in clone R17-12 C1. Cytoscape free software was used to represent the proteins connected with their main predicted functions. The colours were based on the n-fold abundance data in the clone with the highest initial virulence compared with the other clone. For explanation of the abbreviated protein names see cytoscape reference numbers on Table 3.

Among proteins identified as more abundant in the clone with the highest initial virulence score (R17-12 C1) (Table 3 and Fig 5) are orthologes of two proteases, the immuno-dominant variable surface antigen-like protease, and the GP63-like protein. These proteases were shown to be involved in cell adhesion of other protozoa and pointed out as virulence factors [17, 22]. In addition, two other proteases have been found more abundant in the clone with the highest initial virulence, Clan SB family S8 subtilisin-like serine peptidase and Clan CA family C2 calpain-like cysteine peptidase. Another important group of identified peptides matched with proteins that belong to the cytoskeleton, like alpha-tubulin, Tctex-1 family protein and the erythrocyte membrane-associated giant protein antigen 332 containing protein. Also, proteins of the Ras superfamily or other components acting in signal transduction were more abundant in the clone with the highest initial virulence score. This includes RhoGAP domain containing protein, ARF GAP-like zinc finger-containing protein, Ras family protein and RhoGEF domain containing protein. Three calmodulins were also recognized: EF hand family protein, putative calmodulin and putative centrin. Two of the identified proteins potentially participate in membrane trafficking, the C2 domain containing protein and Vps52 / Sac2 family protein, and other three proteins take part in vesicle-mediated transport (endocytosis and exocytosis): putative SNARE protein, CMGC family protein kinase and SacI homology domain containing protein. It is also important to mention the presence of two putative malate dehydrogenases, which are related to the carbohydrate metabolic process and are also suggested to be virulence factors [18, 23]. Finally, there are proteins involved in carbohydrate and metabolic processes, DNA replication and translation that were more abundant in clone R17-12 C1.

**Table 3:** Proteins more abundant in R17.

Category	Predicted Functions	Description (cn) ^1^	P^2^ (n)^3^	Score^4^	Clone P178-13 C7	Clone R17-12 C1	Fold Change	ANOVA p Value	Accession Number
Cell adhesion	Cell adhesion Protease	Immuno-dominant variable surface antigen-like [*Trichomonas vaginalis* G3] (1)	3 (3)	53,94	4,89E+04	9,28E+05	18,96	2,95E-04	gi|12191712
Cell adhesion	Cell adhesion Protease	GP63-like [*Trichomonas vaginalis* G3] (2)	2 (2)	38,73	6,63E+04	5,16E+05	7,78	3,85E-07	gi|121892295
Cellular metabolism	Cell adhesion Cytoskeleton Protein metabolism	Armadillo/beta-catenin-like repeat family protein [*Trichomonas vaginalis* G3] (3)	2 (2)	26,7	1,56E+04	7,89E+04	5,05	2,14E-08	gi|121911734
Cellular component	Cytoskeleton	alpha-tubulin [*Trichonympha agilis*] (4)	6 (4)	481,79	4,81E+04	2,01E+05	4,18	4,92E-03	gi|496528628
Cellular component	Cytoskeleton	alpha-tubulin 2, partial [*Tritrichomonas foetus*] (5)	3 (1)	115,83	5022,1	1,79E+04	3,57	9,13E-05	gi|33465441
Cellular component	Cytoskeleton Vesicle mediated transport	Tctex-1 family protein [*Trichomonas vaginalis* G3] (6)	4 (4)	142,29	8039,24	2,53E+04	3,15	1,17E-06	gi|121902586
Cellular component	Cytoskeleton Rho GTPase binding Calmodulin binding	Erythrocyte membrane-associated giant protein antigen 332 containing protein [*Trichomonas vaginalis* G3] (7)	3 (3)	58,8	1,48E+04	1,51E+05	10,23	7,82E-08	gi|121905491
Signal transduction	Small GTPase regulator Cytoskeletal protein	RhoGAP domain containing protein [*Trichomonas vaginalis* G3] (8)	2 (2)	32,11	1,10E+05	5,12E+05	4,68	1,22E-05	gi|121898182
Signal transduction	Small GTPase regulator	ARF GAP-like zinc finger-containing protein [*Trichomonas vaginalis* G3] (9)	2 (2)	51,31	8180,38	5,29E+04	6,47	3,75E-06	gi|121912557
Signal transduction	Small GTPase regulator	RhoGEF domain containing protein [*Trichomonas vaginalis* G3] (10)	2 (2)	37,87	6003,78	3,60E+04	5,99	1,62E-05	gi|121917299
Signal transduction	Small GTPase regulator	Ras family protein [*Trichomonas vaginalis* G3] (11)	4 (4)	273,64	3,51E+04	1,33E+05	3,8	4,79E-08	gi|121900431
Cellular metabolism	Calcium signal modulators	EF hand family protein [*Trichomonas vaginalis* G3] (12)	2 (2)	70,33	2,55E+04	1,03E+05	4,06	4,41E-07	gi|121885510
Cellular metabolism	Calcium signal modulators	calmodulin, putative [*Trichomonas vaginalis* G3] (13)	3 (3)	202,21	1,01E+04	3,40E+04	3,36	5,73E-07	gi|121905996
Cellular metabolism	Calcium signal modulators	centrin, putative [*Trichomonas vaginalis* G3] (14)	3 (3)	83,18	1,15E+04	3,79E+04	3,29	1,69E-05	gi|121913299
Cellular metabolism	Membrane trafficking Signal transduction	C2 domain containing protein [*Trichomonas vaginalis* G3] (15)	2 (2)	36,07	7828,24	3,91E+04	4,99	5,52E-03	gi|121897227
Cellular metabolism	Membrane trafficking Rab GTPase binding Deaminase	Vps52 / Sac2 family protein [*Trichomonas vaginalis* G3] (16)	2 (2)	29,81	4624,93	3,38E+04	7,31	1,77E-06	gi|121902006
Exocytosis	Vesicle mediated transport	SNARE protein, putative [*Trichomonas vaginalis* G3] (17)	2 (2)	94,78	1819,33	6479,78	3,56	2,51E-03	gi|121914867
Exocytosis	Vesicle-mediated transport Signal transduction	CMGC family protein kinase [*Trichomonas vaginalis* G3] (18)	2 (2)	48,38	2,85E+04	1,00E+05	3,51	1,94E-03	gi|121882186
Endocytosis	Vesicle mediated transport	SacI homology domain containing protein [*Trichomonas vaginalis* G3] (19)	2 (2)	35,91	7081,01	8,59E+04	12,12	1,50E-05	gi|121895312
Cellular metabolism	Carbohydrate metabolic process	Malate dehydrogenase: SUBUNIT = A, putative [*Trichomonas vaginalis* G3] (20)	4 (4)	245,34	2,16E+05	1,41E+06	6,54	5,90E-06	gi|121896029
Cellular metabolism	Carbohydrate metabolic process	Malate dehydrogenase, putative [*Trichomonas vaginali*s G3] (21)	3 (3)	156,4	1,55E+05	5,36E+05	3,46	2,12E-05	gi|121915536
Cellular metabolism	Carbohydrate metabolic process Energetic metabolism	Starch binding domain containing protein [*Trichomonas vaginalis* G3] (22)	3 (3)	79,81	1,98E+04	1,33E+05	6,73	8,92E-04	gi|121903627
Cellular metabolism	Carbohydrate metabolic process Energetic metabolism	4-alpha-glucanotransferase family protein [*Trichomonas vaginalis* G3] (23)	10 (10)	425,98	4,24E+04	1,30E+05	3,07	6,46E-07	gi|121895827
Cellular metabolism	Carbohydrate metabolic process Transferase-kinase	pyruvate, phosphate dikinase family protein [*Trichomonas vaginalis* G3] (24)	9 (9)	562,62	1,32E+04	9,12E+04	6,9	1,36E-08	gi|121904065
Cellular metabolism	Carbohydrate metabolic process Glycolysis Transferase-kinase	PEP-utilizing enzyme, TIM barrel domain containing protein [*Trichomonas vaginalis* G3] (25)	2 (2)	74,26	1978,28	2,01E+04	10,16	1,22E-03	gi|121914977
Cellular metabolism	Carbohydrate metabolic process Glycolysis	enolase 4, putative [*Trichomonas vaginalis* G3] (26)	3 (2)	88,69	5589,54	2,28E+04	4,07	4,60E-06	gi|121908406
Cellular metabolism	Nucleobase-containing compound metabolic process Glycogen metabolic process Response to stress Transcription Metallophosphatase	phosphatase, putative [*Trichomonas vaginalis* G3] (27)	3 (3)	110,32	6005,06	2,02E+04	3,37	2,59E-08	gi|121888987
Cellular metabolism	Energetic metabolism Nucleobase-containing compound metabolic process	Phosphorylase family protein [*Trichomonas vaginalis* G3] (28)	3 (3)	94,45	9127,21	2,96E+04	3,24	1,04E-04	gi|121891200
Cellular metabolism	Peptidase	Clan SB, family S8, subtilisin-like serine peptidase [*Trichomonas vaginalis* G3] (29)	2 (2)	47,09	1,63E+04	9,69E+04	5,93	3,44E-04	gi|121885232
Cellular metabolism	Peptidase Cysteine protease Calmodulin	Clan CA, family C2, calpain-like cysteine peptidase [*Trichomonas vaginalis* G3] (30)	3 (3)	44,02	3,07E+04	1,50E+05	4,88	3,37E-04	gi|121907418
Cellular metabolism	Oxidoreductase-peroxidase Response to stress	hybrid-cluster protein, putative [*Trichomonas vaginalis* G3] (31)	2 (2)	32,17	1467,22	2,69E+04	18,33	8,26E-06	gi|121908608
Cellular metabolism	Protein metabolism Response to stress Chaperone	DnaJ domain containing protein [*Trichomonas vaginalis* G3] (32)	2 (2)	61,26	2597,33	2,66E+04	10,22	6,84E-05	gi|121911862
Cellular metabolism	Protein synthesis Transcription factor	NAC domain containing protein [*Trichomonas vaginalis* G3] (33)	2 (2)	125,14	2206,15	6,49E+04	29,42	2,64E-06	gi|121897587
Cellular metabolism	Protein synthesis Translation	ribosomal protein L10a [*Trichomonas vaginalis*] (34)	2 (2)	136,77	4,43E+04	2,00E+05	4,52	2,10E-04	gi|4193363
Cellular metabolism	Protein synthesis	elongation factor 1 alpha, partial [*Trichomonas vaginalis*] (35)	11 (11)	526,1	4,75E+05	1,44E+06	3,02	9,55E-05	gi|329750813
Cellular metabolism	Protein-protein interaction Ribosyltransferase	B-box zinc finger family protein [*Trichomonas vaginalis* G3] (36)	2 (2)	30,34	1,00E+04	5,62E+04	5,61	4,39E-09	gi|121906335
Cellular metabolism	Protein-protein interaction	erythrocyte binding protein, putative [*Trichomonas vaginalis* G3] (37)	4 (4)	181,54	2,41E+04	1,26E+05	5,22	1,63E-08	gi|121898736
DNA replication	DNA replication	XRN 5'-3' exonuclease N-terminus family protein [*Trichomonas vaginalis* G3] (38)	2 (2)	38,94	3998,07	2,45E+04	6,13	2,43E-04	gi|121913977
DNA replication	DNA replication DNA recombination and DNA repair Response to stress	ATP-dependent DNA helicase, RecQ family protein [*Trichomonas vaginalis* G3] (39)	2 (2)	34,18	1,04E+04	3,74E+04	3,59	2,96E-04	gi|121909374
DNA replication	Nucleus regulation Chromatin replication	SNF2 family N-terminal domain containing protein [*Trichomonas vaginalis* G3] (40)	2 (2)	28,25	1,23E+05	9,67E+05	7,86	1,25E-05	gi|121903240
Cellular component	Translation	SRP54-type protein, putative [*Trichomonas vaginalis* G3] (41)	4 (4)	103,41	3,00E+04	1,48E+05	4,91	3,56E-03	gi|121902866
Translation	Translation	eukaryotic release factor 3 GTPase subunit [*Trichomonas vaginalis*] (42)	3 (3)	116,13	3470,55	1,83E+04	5,27	7,97E-04	gi|8307949
Translation	Translation	translation initiation factor eIF-5A family protein [*Trichomonas vaginalis* G3] (43)	2 (2)	108,14	6,24E+04	2,49E+05	3,99	2,43E-05	gi|121876621

Identified and relatively quantified proteins from membrane proteins enriched fraction for *T*. *gallinae* clonal cultures more abundant in clone R17-12 C1. Proteins were grouped by category, protein function and fold change. Only proteins with 2 or more identified peptides, ≥ 3 fold change and statistical significance (ANOVA, *p*-value < 0.01) are listed.

^**1**^ cn: cytoscape reference number in Fig 4.

^**2**^ P: number of identified peptides.

^**3**^ (n): number of unique, non-conflicting peptides.

^**4**^ total protein score (sum of individual peptides scores).

**Fig 6:**
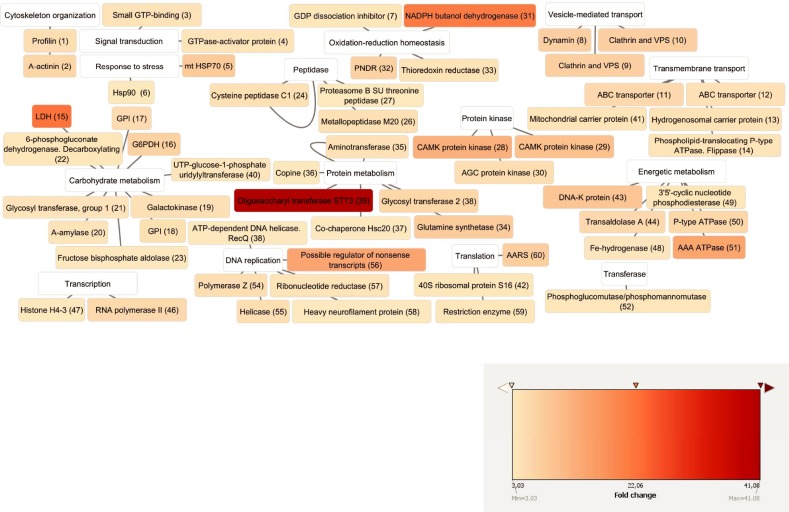
Proteins more abundant in P178. Proteins more abundant in clone P178-13 C7. Cytoscape free software was used to represent the proteins connected with their main predicted functions. The colours were based on the n-fold abundance data in the clone with the lowest initial virulence compared with the other clone. For explanation of the abbreviated protein names see cytoscape reference number on Table 4.

In the clone with the lowest initial virulence (P178-13 C7), proteins associated with stress response, such as HSP 70 and HSP 90, were identified (Table 4 and Fig 6). Only two of the proteins were related to the cytoskeleton: putative profilin and putative alpha-actinin. Two of the proteins which were found more abundant in this clone participate in signal transduction: the putative small GTP-binding protein and GTPase-activator protein. Another group of identified proteins was associated with vesicle-mediated transport (endocytosis and exocytosis) and transmembrane transport: GDP dissociation inhibitor family protein, Dynamin central region family protein, Clathrin and VSP domain-containing protein, ABC transporter family protein, hydrogenosomal carrier protein and phospholipid-translocating P-type ATPase, flippase family protein. A large group of proteins that were also more abundant in the clone with the lowest initial virulence score were related to cellular metabolism, more precisely carbohydrate metabolism. Among them, lactate dehydrogenase family protein, glucose-6-phosphate 1-dehydrogenase family protein, glucose-6-phosphate isomerase family protein, galactokinase family protein, alpha amylase, catalytic domain containing protein, glycosil transferase group 1 family protein, 6-phosphogluconate dehydrogenase decarboxylating family protein and putative fructose 1,6-bisphosphate aldolase, can be mentioned. Also, the cysteine peptidase Clan CA, family C1, cathepsin L-like and the metalloprotease Clan MH, family M20, peptidase T-like were more abundant in this clone. Finally, a group of proteins implied in protein metabolic processes, DNA replication, translation and translocation were also identified as more abundant in clone P178-13 C7.

**Table 4:** Proteins more abundant in P178.

Category	Predicted Functions	Description (cn)^1^	P^2^ (n)^3^	Score^4^	Clone P178-13 C7	Clone R17-12 C1	Fold Change	ANOVA p Value	Accession Number
Cellular component	Cytoskeleton	profilin, putative [*Trichomonas vaginalis* G3] (1)	2 (2)	64,27	6,67E+04	1,40E+04	4,75	2,17E-05	gi|121906940
Cellular component	Cytoskeleton Metal ion binding	putative alpha-actinin [*Histomonas meleagridis*] (2)	3 (3)	48,12	7720,57	1239	6,23	2,59E-03	gi|262358541
Signal transduction	Signal transduction	small GTP-binding protein, putative [*Trichomonas vaginalis* G3] (3)	2 (2)	237,3	3,06E+05	7,28E+04	4,21	1,97E-03	gi|121880468
Signal transduction	Signal transduction	GTPase-activator protein, putative [*Trichomonas vaginalis* G3] (4)	2 (2)	29,36	2,40E+05	6,07E+04	3,95	3,09E-04	gi|121892626
Cellular metabolism	Response to stress	Heat shock 70 kDa protein, mitochondrial precursor, putative [*Trichomonas vaginalis* G3] (5)	2 (2)	74,39	2,02E+05	2,48E+04	8,13	1,66E-03	gi|121914923
Cellular metabolism	Response to stress	Hsp90 protein [*Trichomonas vaginalis* G3] (6)	6 (6)	326,25	3,83E+04	1,19E+04	3,22	1,97E-05	gi|121917639
Cellular metabolism	Oxidation-reduction homeostasis Vesicle-mediated transport Small GTPase regulator (-) Signal transduction	GDP dissociation inhibitor family protein [*Trichomonas vaginalis* G3] (7)	6 (6)	355,73	1,37E+05	3,96E+04	3,46	4,89E-05	gi|121879717
Endocytosis	Vesicle-mediated transport Cytoskeleton Small GTPase	Dynamin central region family protein [*Trichomonas vaginalis* G3] (8)	3 (3)	64,69	5,79E+04	7821,44	7,4	5,98E-05	gi|121887114
Exocytosis	Vesicle-mediated transport	Clathrin and VPS domain-containing protein, partial [*Trichomonas vaginalis* G3] (9)	8 (8)	349,57	1,08E+05	1,51E+04	7,17	1,06E-04	gi|121873597
Exocytosis	Vesicle-mediated transport	Clathrin and VPS domain-containing protein [*Trichomonas vaginalis* G3] (10)	6 (6)	208,37	1,46E+05	2,06E+04	7,11	2,48E-05	gi|121898439
Cellular metabolism	Transmembrane transport	ABC transporter family protein [*Trichomonas vaginalis* G3] (11)	4 (4)	149,49	2,76E+04	5066,61	5,45	3,12E-07	gi|121883001
Cellular metabolism	Transmembrane transport	ABC transporter family protein [*Trichomonas vaginalis* G3] (12)	3 (3)	99,04	3,27E+04	7707,22	4,24	7,94E-05	gi|121889755
Cellular metabolism	Transmembrane transport	hydrogenosomal carrier protein, partial [*Trichomonas gallinae*] (13)	14 (14)	744,55	2,07E+06	5,02E+05	4,12	5,36E-04	gi|30315255
Cellular metabolism	Transmembrane transport	phospholipid-translocating P-type ATPase, flippase family protein [*Trichomonas vaginalis* G3] (14)	2 (2)	49,93	1,47E+05	3,84E+04	3,81	1,56E-05	gi|121897029
Cellular metabolism	Carbohydrate metabolic process Energetic metabolism	lactate dehydrogenase family protein [*Trichomonas vaginalis* G3] (15)	2 (2)	43,1	1,21E+05	5473,69	22,13	3,85E-07	gi|121889193
Cellular metabolism	Carbohydrate metabolic process Energetic metabolism	glucose-6-phosphate 1-dehydrogenase family protein [*Trichomonas vaginalis* G3] (16)	7 (7)	285,86	8,47E+04	1,21E+04	7	2,95E-07	gi|121883413
Cellular metabolism	Carbohydrate metabolic process Gluconeogenesis Glycolysis	glucose-6-phosphate isomerase family protein [*Trichomonas vaginalis* G3] (17)	17 (17)	917,8	2,76E+05	5,44E+04	5,08	1,37E-04	gi|121906378
Cellular metabolism	Carbohydrate metabolic process Energetic metabolism	glucose-6-phosphate isomerase family protein [*Trichomonas vaginalis* G3] (18)	3 (3)	89,42	7,67E+04	1,71E+04	4,49	1,22E-06	gi|121908707
Cellular metabolism	Carbohydrate metabolic process Energetic metabolism	galactokinase family protein [*Trichomonas vaginalis* G3] (19)	3 (3)	134,69	2,73E+04	6100,32	4,47	3,28E-07	gi|121903545
Cellular metabolism	Carbohydrate metabolic process Energetic metabolism	Alpha amylase, catalytic domain containing protein [*Trichomonas vaginalis* G3] (20)	2 (2)	94,07	1,35E+04	3431,11	3,94	9,70E-04	gi|121915021
Cellular metabolism	Carbohydrate metabolic process Energetic metabolism	glycosyl transferase, group 1 family protein [*Trichomonas vaginalis* G3] (21)	2 (2)	43,52	2,36E+04	6251,76	3,78	5,41E-05	gi|121889369
Cellular metabolism	Carbohydrate metabolic process Energetic metabolism	6-phosphogluconate dehydrogenase, decarboxylating family protein [*Trichomonas vaginalis* G3] (22)	2 (2)	99,56	1,10E+05	3,03E+04	3,64	6,83E-06	gi|121896682
Cellular metabolism	Carbohydrate metabolic process Metal ion binding Lyase	fructose-1,6-bisphosphate aldolase, putative [*Trichomonas vaginalis* G3] (23)	12 (12)	714,5	1,64E+06	5,03E+05	3,26	2,12E-03	gi|121909882
Cellular metabolism	Protein metabolism cysteine peptidase	Clan CA, family C1, cathepsin L-like cysteine peptidase [*Trichomonas vaginalis* G3] (24)	2 (2)	43,69	2,27E+04	4950,94	4,59	3,18E-03	gi|121908100
Cellular metabolism	Protein metabolism Peptidase	Clan CA, family C1, cathepsin L-like cysteine peptidase, partial [*Trichomonas gallinae*] (25)	6 (6)	286,99	1,28E+06	3,49E+05	3,67	1,69E-03	gi|386364444
Cellular metabolism	Protein metabolism metalloprotease	Clan MH, family M20, peptidase T-like metallopeptidase [*Trichomonas vaginalis* G3] (26)	3 (3)	123,12	1,34E+05	2,97E+04	4,49	1,24E-04	gi|121901617
Cellular metabolism	Protein metabolism threonine peptidase	Family T1, proteasome beta subunit, threonine peptidase [*Trichomonas vaginalis* G3] (27)	3 (3)	94,65	3,75E+04	1,24E+04	3,03	1,47E-04	gi|121916758
Cellular metabolism	Protein metabolism protein kinases	CAMK family protein kinase [*Trichomonas vaginalis* G3] (28)	2 (2)	31,32	8977,61	715,69	12,54	5,04E-03	gi|121906466
Cellular metabolism	Protein metabolism protein kinases	CAMK family protein kinase [*Trichomonas vaginalis* G3] (29)	2 (2)	32,25	1,62E+04	1619,25	9,98	1,01E-03	gi|121912207
Cellular metabolism	Protein metabolism Protein kinase Signal transduction	AGC family protein kinase [*Trichomonas vaginalis* G3] (30)	2 (2)	54,75	1,69E+05	3,65E+04	4,63	9,97E-03	gi|121890243
Cellular metabolism	Metal ion binding Oxidation-reduction homeostasis	putative NADPH-dependent butanol dehydrogenase [*Trichomonas vaginalis*] (31)	2 (2)	206,35	4,42E+04	2102,87	21,01	2,18E-05	gi|4426910
Cellular metabolism	Metal ion binding Oxidation-reduction homeostasis	Pyridine nucleotide-disulphide oxidoreductase family protein [*Trichomonas vaginalis* G3] (32)	3 (3)	178,07	2,07E+05	3,00E+04	6,9	9,64E-08	gi|121898098
Cellular metabolism	Oxidation-reduction homeostasis	thioredoxin-disulfide reductase family protein [*Trichomonas vaginalis* G3] (33)	2 (2)	113,87	1,21E+05	2,93E+04	4,14	1,62E-03	gi|121893127
Cellular metabolism	Protein metabolism	Glutamine synthetase, catalytic domain containing protein [*Trichomonas vaginalis* G3] (34)	3 (3)	107,13	7,43E+04	9390,65	7,91	1,67E-08	gi|121892813
Cellular metabolism	Protein metabolism	aminotransferase, classes I and II family protein [*Trichomonas vaginalis* G3] (35)	2 (2)	164,19	1,55E+05	4,54E+04	3,41	3,92E-05	gi|121909674
Cellular metabolism	Protein metabolism	Copine family protein [*Trichomonas vaginalis* G3] (36)	3 (3)	53,44	6,76E+04	2,14E+04	3,16	2,31E-03	gi|121901567
Cellular metabolism	Protein metabolism	co-chaperone Hsc20 family protein [*Trichomonas vaginalis* G3] (37)	2 (2)	70,46	3,19E+04	1,03E+04	3,11	0,01	gi|121889907
Cellular metabolism	Protein metabolism Phospholipid metabolic process	glycosyl transferase, group 2 family protein [*Trichomonas vaginalis* G3] (38)	4 (4)	195,85	2,82E+04	4751,28	5,94	1,67E-05	gi|121911637
Cellular metabolism	Energetic metabolism Protein glycosylation	Oligosaccharyl transferase STT3 subunit family protein [*Trichomonas vaginalis* G3] (39)	2 (2)	63,86	1,33E+04	323,32	41,08	7,69E-08	gi|121902670
Cellular metabolism	Glycogen metabolic process Transferase	UTP-glucose-1-phosphate uridylyltransferase family protein [*Trichomonas vaginalis* G3] (40)	5 (5)	168,95	2,17E+04	6092,98	3,56	2,44E-06	gi|121904554
Cellular metabolism	Translation Transmembrane transport Calmodulin Ribosomal protein Mitocondrial carrier protein	Mitochondrial carrier protein [*Trichomonas vaginalis* G3] (41)	9 (9)	479,27	6,94E+05	1,77E+05	3,92	4,36E-04	gi|121889868
Cellular component	Ribosomal protein RNA metabolic process Protein metabolic processes	40S ribosomal protein S16, putative [*Trichomonas vaginalis* G3] (42)	2 (2)	143,1	5,03E+04	1,45E+04	3,47	7,03E-04	gi|121885138
Cellular metabolism	Energetic metabolism Protein complex assembly HSP70/actin superfamily	dnaK protein [*Trichomonas vaginalis* G3] (43)	4 (4)	101,55	1,64E+05	1,84E+04	8,93	5,84E-08	gi|121907663
Cellular metabolism	Transaldolase	Transaldolase A, putative [*Trichomonas vaginalis* G3] (44)	2 (2)	62,98	2,40E+04	4347,89	5,52	8,56E-05	gi|121896456
Cellular metabolism	Transaminase Cellular aminoacid metabolic process	aminotransferase, classes I and II family protein [*Trichomonas vaginalis* G3] (45)	3 (3)	158,74	1,17E+05	2,72E+04	4,3	9,78E-03	gi|121901152
Cellular metabolism	Transcription	RNA polymerase II largest subunit, partial [*Trichomonas gallinae*] (46)	2 (2)	87,66	8257,66	1452,08	5,69	2,60E-04	gi|327242119
Cellular metabolism	Transcription	histone H4-3 [*Trichomonas vaginalis* G3] (47)	2 (2)	184,78	2,99E+05	9,01E+04	3,32	3,19E-03	gi|121889898
Cellular component	Hydrogenase, metal ion binding	hydrogenosomal Fe-hydrogenase, partial [*Trichomonas gallinae*] (48)	10 (10)	704,23	2,57E+05	7,19E+04	3,58	8,81E-03	gi|507310429
Cellular metabolism	Energetic metabolism Nucleobase containing compound metabolic process Signal transduction	3'5'-cyclic nucleotide phosphodiesterase family protein [*Trichomonas vaginalis* G3] (49)	3 (3)	60,29	4,57E+05	1,29E+05	3,54	7,28E-04	gi|121889147
Cellular metabolism	ATPase Calcium signal modulators Nucleobase-containing compound metabolic process Transmembrane calcium ion transport	calcium-translocating P-type ATPase, PMCA-type family protein [*Trichomonas vaginalis* G3] (50)	2 (2)	71,75	5,25E+04	8685,17	6,04	2,13E-05	gi|121889617
Cellular metabolism	ATPase	ATPase, AAA family protein [*Trichomonas vaginalis* G3] (51)	2 (2)	30,29	5,54E+05	3,85E+04	14,38	1,45E-06	gi|121886218
Cellular metabolism	Metal ion binding Transferase	Phosphoglucomutase/phosphomannomutase, alpha/beta/alpha domain I family protein [*Trichomonas vaginalis* G3] (52)	8 (8)	414,71	2,67E+05	7,70E+04	3,47	3,13E-05	gi|121885206
Cellular metabolism	DNA replication Energetic metabolism	ATP-dependent DNA helicase, RecQ family protein [*Trichomonas vaginalis* G3] (53)	3 (2)	54,81	2,05E+05	6,37E+04	3,22	2,82E-03	gi|121888663
DNA replication	DNA replication	polymerase zeta subunit, putative [*Trichomonas vaginalis* G3] (54)	2 (2)	36,13	2,96E+04	6239,31	4,74	1,58E-06	gi|121909895
DNA replication	DNA replication	helicase, putative [*Trichomonas vaginalis* G3] (55)	2 (2)	36,9	4,43E+04	9427,08	4,7	2,77E-07	gi|121911876
DNA replication	DNA replication Transcription	possible regulator of nonsense transcripts, putative [*Trichomonas vaginalis* G3] (56)	3 (3)	81,75	4,87E+05	3,46E+04	14,09	7,25E-07	gi|121916893
DNA replication	DNA replication Transcription	Ribonucleotide reductase, all-alpha domain containing protein [*Trichomonas vaginalis* G3] (57)	5 (5)	255,06	1,61E+05	3,94E+04	4,09	2,53E-04	gi|121915211
DNA replication	Nucleotide binding	heavy neurofilament protein, putative [*Trichomonas vaginalis* G3] (58)	3 (3)	46,16	1,54E+04	4756,37	3,23	5,43E-05	gi|121898592
Translation	RNA metabolic process	Type III restriction enzyme, res subunit family protein [*Trichomonas vaginalis* G3] (59)	2 (2)	46,03	7,36E+05	2,01E+05	3,66	8,63E-03	gi|121898850
Translation	Metal ion binding	alanyl-tRNA synthetase, partial [*Trichomonas vaginalis*] (60)	5 (5)	187,01	1,31E+05	1,71E+04	7,63	8,44E-06	gi|51102334

Identified and relatively quantified proteins from membrane proteins enriched fraction for *T*. *gallinae* clonal cultures more abundant in clone P178-13 C7. Proteins were grouped by category, protein function and fold change. Only proteins with 2 or more identified peptides, ≥ 3 fold change and statistical significance (ANOVA, *p*-value < 0.01) are listed.

^**1**^ cn: cytoscape reference number in Fig 5

^**2**^ P: number of identified peptides.

^**3**^ (n): number of unique, non-conflicting peptides.

^**4**^ total protein score (sum of individual peptides scores).

## Discussion

Considering that mixed infections have been detected during the natural course of the *T*. *gallinae* infection, the use of clonal cultures, established by micromanipulation, guarantees genetic homogeneity of the investigated parasite [3, 4, 6, 9, 11]. This is crucial in order to perform detailed studies on the parasite’s biology, especially the proteome.

The employment of cell cultures to determine the pathogenicity of *T*. *gallinae* isolates offers an *in vitro* approach in addition to animal trials. Some of the variables that can interfere with the results, such as the individual immune response of the animals, are not influencing the *in vitro* system and can therefore easier be standardized as well as better controlled in its variability. Differences in virulence between the clones were detected at 24 and 48 hours post-infection and were associated with the parasite genotype. Amin et al. [14] also observed variation in the CPE score of the *T*. *gallinae* clones employed in their study, in which one clone of genotypic group ITS-OBT-Tg-1 caused complete cell monolayer destruction whilst a ITS-OBT-Tg-2 clone induced only 50% of destruction. These results are in agreement with the observations from natural infections in some studies carried out in Europe [6, 11–13].

The membrane proteins enriched fraction of the parasite was selected for further proteomic characterization due to the potential role of the membrane proteins in the process of adhesion to host cells and signal trafficking. The adhesion is important to maintain the colonization of virulent protozoa, but also of less virulent ones. The role of membranes proteins in the establishment of infection has already been demonstrated with other protozoa, such as *Giardia duodenalis* and *Entamoeba histolytica* [31, 32]. Some of the identified and quantified proteins, such as enolase, have been also identified as moonlighting proteins with double functions in other protozoa, like *Toxoplasma*, *Trypanosoma* or *Trichomonas* spp. and for that reason they could have been found in different subcellular locations [33]. Vesicular trafficking is extensive in the membrane system, favoring the translocation of proteins along the cell compartments.

Among the proteins that differed in abundance, a large amount of proteins with no assigned function was revealed. This result was expected, as many of the membranes proteins are specific for each microorganism and are possibly implicated in host-parasite species-specific interactions [19].

Colonization of the host is a first step necessary for either commensal or pathogenic microorganisms [34]. In this sense, the identification of peptides from different surface proteins of the Leucine Rich Repeat family proteins and surface antigen BspA-like, with different abundance in both clones, might be related to the colonization of the mucosa and the establishment of a chronic infection, but not necessarily with virulence. These proteins are commonly present in a broad range of organisms, including all three domains of life (Eukaryota, Archae and Bacteria), all of them residing on mucosal surfaces or body cavities [17].

Some proteases or other molecules involved in cell adhesion, like the immuno-dominant variable surface antigen-like or the GP63-like, which are considerably more abundant in the clone with the highest initial virulence score (18.96 and 7.78 fold change, respectively), have been suggested as virulence factors in other protozoa, like *T*. *vaginalis* and *Leishmania* sp.. They act by binding and degrading various host components [17, 22]. In *T*. *vaginalis* a protein of the GP63-like group was determined to be involved in the virulence on HeLa cells [20]. The presence of the armadillo/beta-catenin-like repeat family protein in a higher amount in the clone with the highest initial virulence score also indicates that these cell adhesion molecules could be important in the pathogenesis of avian trichomonosis. Eukaryotic armadillo repeat proteins (beta-catenin-like molecules) usually play a role in cell-to-cell adhesion and cytoskeletal regulation together with intracellular signaling. They have been identified in some apicomplexan parasites, probably acting during the invasion process [35]. Malate dehydrogenase has been found more abundant in the clone with the highest initial virulence score and it could be a virulence factor since large amounts of malate dehydrogenase were found in amoeboid trophozoites of *T*. *vaginalis* bound to fibronectin, suggesting the relationship with the adherence of the parasite to the host cells [23]. Some forms of the malate dehydrogenase were also exclusively expressed in a fresh clinical isolate of *T*. *vaginalis*, (high virulence) in comparison with a long-term culture (low virulence) [18].

Although several proteases have been identified as more abundant in one or the other clone, a Clan SB family S8 subtilisin-like serine peptidase has been identified as more abundant in the clone with the highest initial virulence score. This protease has been found in higher amounts in adherent isolates of the closely related protozoan *T*. *vaginalis* [19]. Clan CA family C2 calpain-like cysteine peptidase, which is more abundant in the clone with the highest initial virulence score, has also been pointed out as a potential virulence factor in *T*. *vaginalis* [17]. On the other hand, Clan CA family C1 cathepsin L-like cysteine peptidases were more abundant in the clone with the lowest initial virulence score. Interestingly, one of these peptidases was shown to be responsible for the cytopathogenic effect of *T*. *gallinae* in permanent LMH cell cultures, however the approach (gel-based proteomics) and the analyzed protein sample (cell-free extract which corresponded to secreted proteins) employed in that study were different which might have contributed to the different result [15].

It has been proposed that *T*. *vaginalis* is able to bind to epithelial cells before transforming into an amoeboid form to increase the cell-to-cell surface contact. This fact implies a high variability of the cytoskeletal proteins [19, 23]. Our results indicate that a similar mechanism could occur in *T*. *gallinae*. Several peptides of the Ras superfamily small GTPases or components involved in their activation, like RhoGAP domain containing protein, the ARF GAP-like zinc finger containing protein; together with the Ras family protein and RhoGEF domain containing protein, have been identified as more abundant in the clone with the highest initial virulence score. In the clone with the lowest initial virulence score, only peptides mapping to two proteins related with this group were found in higher amounts: the putative small GTP-binding protein and the putative GTPase-activator protein. Overall, small GTPases and components involved in their activation are related to cell differentiation, proliferation (Ras subfamily), cytoskeletal organization, cell motility and oxidative stress (Rho subfamily), vesicle formation and regulation of vesicular trafficking (Rab, ARF and Sar subfamilies) and cell signaling in protozoa [36]. In *E*. *histolytica*, Rho GTPases, RhoGEFs and Rho effectors regulate the dynamic actin cytoskeleton and are associated with pathogenesis-related processes, such as invasion, migration, phagocytosis and evasion of the host immune response by surface receptor capping. Furthermore, Rab GTPases participate in phagocytosis as well as pinocytosis by the vesicular trafficking system and in the secretion of virulence factors, like amebapores and cysteine proteases in *E*. *histolytica* [32, 37]. In connection with the Ras superfamily proteins, it is important to note that peptides from a group of proteins involved in cytoskeleton organization, like Tctex-1 family protein or the erythrocyte membrane-associated giant protein antigen 332 containing protein, were more abundant in the membrane of the clone with the highest initial virulence score. These proteins interact with Rho subfamily proteins for regulation of the cytoskeleton re-organization. Also, two putative alpha tubulins, components of the cytoskeleton, were identified as more abundant in this clone. In the clone with delayed virulence, we only found higher amounts of peptides from two cytoskeletal proteins, in a putative profilin and a putative alpha actinin, indicating that re-organization of the cytoskeleton might be one of the mechanisms facilitating the virulence. However, more detailed studies are necessary to confirm this.

It is interesting to note that peptides from three proteins with calmodulin-like predicted function have been identified as more abundant in the clone with the highest initial virulence score. These proteins can be found in organelle membranes and play a role in the activation of phosphorylase kinases or phosphatases, necessary for cytoskeletal re-organization. Calmodulin is involved in the movement of *Giardia intestinalis* [38] and a calmodulin-like protein has been identified in *E*. *histolytica* playing a role in the phagocytosis of erythrocytes and cytoskeleton dynamics [39].

A group of peptides from proteins implicated in vesicular trafficking, differed in abundance between both clones. In the clone with the highest initial virulence score, peptides from a putative SNARE protein, a CMGC family protein kinase and a SacI homology domain containing protein were more abundant, whereas the opposite situation was observed with dynamin central region family protein and clathrin and vacuolar protein sorting (VPS) domain. All of these proteins are involved in endocytosis or exocytosis processes. The difference in abundance of proteins related to vesicular trafficking might be due to variations in the excretion process or feeding process between both clones including cell phagocytosis, autophagy of other trophozoites and pinocytosis of the culture medium components. Autophagy has been shown to occur in *T*. *vaginalis* cultures during the restriction of nutrients [40]. It is also noteworthy that the large group of proteins (n = 7) associated with the carbohydrate metabolism was more abundant in the clone with the lowest initial virulence score in contrast to only one protein in the clone with the highest initial virulence score. This observation can be related to the higher growth rate of the clone with the lowest initial virulence score in TYM medium as compared to the clone with the highest initial virulence score (S1 Fig).

A difference in abundance of peptides from proteins involved in plasmatic and organelles membrane transport was evident: the C2 domain containing protein and Vps52/Sac2 family protein were more abundant in the clone with the highest initial virulence score, whereas the hydrogenosomal carrier protein, the phospholipid-translocating P-type ATPase flippase family protein and the ABC transporter family protein were more abundant in the clone with the lowest initial virulence score. Interestingly, this result is in agreement with de Miguel et al [19], who identified an ABC transporter protein to be more abundant in less adherent isolates of *T*. *vaginalis*. This fact could also be related to the better growth of the clone with the lowest initial virulence score, since energy processes seem to be more active.

## Conclusions

To the best of our knowledge, this is the first approach to identify and quantify membrane proteins of *T*. *gallinae*. Differences in the abundance of some peptides between the two clones were analyzed, which provides basic knowledge on disparities of strains within the same parasite species. The differences could be related to the distinct genotype, to the variations in their initial virulence or even to both characteristics. Some of the identified proteins in the present study can be potential virulence factors, similar to their orthologs in *T*. *vaginalis* and other pathogenic protozoa. Among them, we can highlight the metalloprotease GP63-like, the immuno-dominant variable surface antigen-like, the group of small GTPase Ras superfamily proteins, the calmodulins, the proteases Clan SB family S8 subtilisin-like serine peptidase and the Clan CA family C2 calpain-like cysteine peptidase. The clone with the highest initial virulence had also a higher amount of proteins related to cell adhesion, cytoskeleton organization and membrane and vesicular trafficking processes. On the contrary, the clone with the lowest initial virulence score showed higher abundance of proteins related to metabolic processes, which might be related to the better growth in the medium. These results point towards possible changes in the trophozoite shape of *T*. *gallinae* in a similar way as the human pathogen *T*. *vaginalis*.

## Supporting information

S1 Fig Growth of P178-13 C7 and R17-12 C1 clones in TYM. Number of live *T*. *gallinae* trophozoites/ml from clonal cultures P178-13 C7 and R17-12 C1 in TYM medium. Error bars indicate standard deviation.
(EPS)

S2 Fig SDS-PAGE profile of clones. SDS-PAGE analysis from the enriched membrane proteins fraction of *T*. *gallinae* clonal cultures P178-13 C7 P11 (lane 2) and R17-12 C1 P9 (lane 1). MW marker (Precision Plus Protein^^™^^ Standards Dual Color de (Biorad, Alcobendas, Spain)) are in line 3.
(EPS)

S3 Fig LC-MS/MS chromatograms. LC-MS/MS chromatograms from the enriched membrane proteins fraction of *T*. *gallinae* clonal cultures P178-13 C7 P11 and R17-12 C1 P9.
(TIF)

S1 Table Hypothetical proteins more abundant in P178. Hypothetical proteins relatively quantified proteins from the enriched membrane proteins fraction for *T*. *gallinae* clonal cultures that are more abundant in clone P178-13 C7 with two or more identified peptides, ≥ 3 fold change and statistical significance (ANOVA, *p*-value < 0.01). P: number of identified peptides. (n): number of unique, non-conflicting peptides. Score: Total protein score (sum of individual peptides scores).
(PDF)

S2 Table Hypothetical proteins more abundant in R17. Hypothetical proteins relatively quantified proteins from the enriched membrane proteins fraction for *T*. *gallinae* clonal cultures that are more abundant in clone R17-12 C1 with 2 or more identified peptides, ≥ 3 fold change and statistical significance (ANOVA, *p*-value < 0.01). P: number of identified peptides. (n): number of unique, non-conflicting peptides. Score: Total protein score (sum of individual peptides scores).
(PDF)

S1 Data Original data of S1 Fig and Fig 2. Original Data used to construct graph of S1 Fig. Original Data used to construct graphs of Fig 2.
(XLSX)
