# Supplementary material for: Membrane associated proteins of two Trichomonas gallinae clones vary with the virulence
Source: PLoS One. 2019 Oct 24;14(10):e0224032. doi: 10.1371/journal.pone.0224032 (PMC6812828; doi:10.1371/journal.pone.0224032)
Supplement: S1 Table — Hypothetical proteins relatively quantified proteins from the enriched membrane proteins fraction for T. gallinae clonal cultures that are more abundant in clone P178-13 C7 with two or more identified peptides, ≥ 3 fold change and statistical significance (ANOVA, p-value < 0.01). P: number of identified peptides. (n): number of unique, non-conflicting peptides. Score: Total protein score (sum of individual peptides scores). (PDF) [file pone.0224032.s004.pdf]

Hypothetical proteins up-regulated in P178. Hypothetical proteins relatively quantified proteins from membrane and organelle membrane fraction for *T. gallinae* clonal cultures that are more abundant in clone P178-13 C7 with two or more identified peptides, ≥ 3 fold change and statistical significance (ANOVA, *p*-value < 0.01). P: number of identified peptides. (n): number of unique, non-conflicting peptides. Score: Total protein score (sum of individual peptides scores).

Predicted Function	Fold Change	Category	Description	P	Score	P178-13	R17-12	*p*-value	Accesion Number
Binding of large substrates	10	Cellular metabolism	hypothetical protein TVAG_109510 [*Trichomonas vaginalis* G3]	2 (2)	29,24	4,60E+05	4,60E+04	3,33E-04	gi|121905439
Binding of large substrates	3,83	Cellular metabolism	hypothetical protein TVAG_028120 [*Trichomonas vaginalis* G3]	2 (2)	30,5	2,67E+04	6960,72	1,76E-04	gi|121907365
Cell adhesion	31,76	Cell adhesion	hypothetical protein TVAG_130090 [Trichomonas vaginalis G3]	2 (2)	32,04	3,18E+04	1000,03	1,82E-03	gi|121915109
Cell adhesion	7,25	Cell adhesion	conserved hypothetical protein [Trichomonas vaginalis G3]	2 (2)	27,97	4,96E+05	6,84E+04	8,08E-06	gi|121890028
Cell adhesion	3,65	Cell adhesion	hypothetical protein TVAG_457560 [Trichomonas vaginalis G3]	2 (2)	28,54	7,44E+04	2,04E+04	3,02E-04	gi|121917345
Cytoskeleton	12,34	Cellular component	hypothetical protein TVAG_208940 [Trichomonas vaginalis G3]	3 (3)	45,13	5,98E+04	4845,66	9,04E-06	gi|121910773
Cytoskeleton	9,25	Cellular component	hypothetical protein TVAG_110590 [Trichomonas vaginalis G3]	4 (4)	54,08	2,93E+04	3163,73	7,36E-05	gi|121915660
Cytoskeleton	8,62	Cellular component	hypothetical protein TVAG_454480 [Trichomonas vaginalis G3]	2 (2)	33,83	1,15E+04	1337,43	4,29E-04	gi|121898727
Cytoskeleton	6	Cellular component	hypothetical protein TVAG_252220 [Trichomonas vaginalis G3]	3 (3)	42,53	2,22E+05	3,70E+04	6,78E-05	gi|121910564
Cytoskeleton	5,74	Cellular component	hypothetical protein TVAG_169860 [Trichomonas vaginalis G3]	3 (3)	54,45	5,49E+04	9577,77	1,16E-04	gi|121912858
Cytoskeleton	5,38	Cellular component	hypothetical protein TVAG_329890 [Trichomonas vaginalis G3]	2 (2)	27,58	9,73E+04	1,81E+04	8,69E-06	gi|121887190
Cytoskeleton	4,16	Cellular component	hypothetical protein TVAG_021520 [Trichomonas vaginalis G3]	2 (2)	40,01	1,35E+05	3,23E+04	6,41E-04	gi|121915416

Cytoskeleton	3,84	Cellular component	hypothetical protein TVAG_477720 [*Trichomonas vaginalis* G3]	2 (2)	45,1	2,78E+05	7,25E+04	4,09E-03	gi|121882914
Vacuole protein	8,21	Cellular component	hypothetical protein TVAG_238570 [Trichomonas vaginalis G3]	3 (3)	45,97	1,82E+05	2,21E+04	4,93E-04	gi|121915699
Metal ion binding	8,23	Cellular metabolism	hypothetical protein TVAG_276700 [*Trichomonas vaginalis* G3]	2 (2)	37,03	2,29E+05	2,79E+04	1,68E-05	gi|121891036
Mitochondrial transport	6,98	Cellular component	hypothetical protein TVAG_548390 [Trichomonas vaginalis G3]	2 (2)	30,34	1,34E+05	1,91E+04	1,35E-04	gi|121841176
Oesophageal cancer-associated protein	19,6	Cell adhesion	hypothetical protein TVAG_008090 [Trichomonas vaginalis G3]	2 (2)	36,49	1,26E+05	6403,58	3,63E-07	gi|121897688
Peptidase	3,23	Cellular metabolism	hypothetical protein TVAG_051850 [Trichomonas vaginalis G3]	2 (2)	43,6	3,61E+04	1,12E+04	1,35E-04	gi|121889871
Phospholipid synthesis	3,07	Cellular metabolism	hypothetical protein TVAG_407170 [Trichomonas vaginalis G3]	6 (6)	348,73	2,85E+05	9,28E+04	2,69E-05	gi|121895175
Protein bindin	5,2	Cellular metabolism	hypothetical protein TVAG_436440 [Trichomonas vaginalis G3]	2 (2)	37,45	6,51E+04	1,25E+04	4,47E-04	gi|121916037
Protein metabolism	6,83	Cellular metabolism	hypothetical protein TVAG_107620 [Trichomonas vaginalis G3]	2 (2)	62,43	2,16E+04	3163,05	2,65E-06	gi|121896349
Protein metabolism	3,01	Cellular metabolism	conserved hypothetical protein [Trichomonas vaginalis G3]	2 (2)	44,55	8215,11	2729,95	5,01E-06	gi|121890884
Protein targeting to Golgi	8,23	Cellular metabolism	hypothetical protein TVAG_201650 [Trichomonas vaginalis G3]	2 (2)	29,22	6770,17	822,38	2,96E-04	gi|121910323
Protein-protein interaction	46,22	Cellular metabolism	hypothetical protein TVAG_147380 [Trichomonas vaginalis G3]	2 (2)	32,88	4,88E+05	1,05E+04	1,01E-05	gi|121880636
Protein-protein interaction	5,62	Cellular metabolism	hypothetical protein TVAG_093700 [Trichomonas vaginalis G3]	3 (3)	41,23	4,05E+04	7208,38	1,75E-06	gi|121917422
Protein-protein interaction	4,06	Cellular metabolism	hypothetical protein TVAG_075500 [Trichomonas vaginalis G3]	2 (2)	31,87	1,96E+05	4,82E+04	1,13E-05	gi|121918033
Ribosomal large subunit assembly	6,02	Cellular component	hypothetical protein TVAG_464510 [Trichomonas vaginalis G3]	2 (2)	37,7	4,56E+04	7570,82	3,91E-06	gi|121903946
Ribosomal large subunit assembly	3,36	Cellular component	hypothetical protein TVAG_470660 [Trichomonas vaginalis G3]	5 (5)	89,09	1,69E+05	5,03E+04	2,62E-05	gi|121901205
Ribosomal large subunit assembly	3,28	Cellular component	hypothetical protein TVAG_476450 [Trichomonas vaginalis G3]	3 (3)	47,7	1,98E+05	6,02E+04	2,32E-03	gi|121917945

RNA processing	7,59	Cellular component	conserved hypothetical protein [Trichomonas vaginalis G3]	2 (2)	56,49	4,09E+04	5381,27	2,58E-06	gi|121899537
Signal transduction	3,67	Signal transduction	hypothetical protein TVAG_530150 [Trichomonas vaginalis G3]	2 (2)	38,53	1,52E+05	4,13E+04	1,15E-04	gi|121877483
Transcription	10,47	Cellular metabolism	hypothetical protein TVAG_285400 [Trichomonas vaginalis G3]	2 (2)	37,86	1,72E+04	1639,41	8,33E-05	gi|121859384
Translation	6,9	Translation	hypothetical protein TVAG_184380 [Trichomonas vaginalis G3]	2 (2)	36,51	3,65E+05	5,29E+04	1,93E-04	gi|121905611
Translation	5,68	Translation	hypothetical protein TVAG_016880 [Trichomonas vaginalis G3]	9 (9)	453,74	1,17E+06	2,06E+05	9,26E-05	gi|121899865
Translation	3,86	Translation	hypothetical protein TVAG_266630 [Trichomonas vaginalis G3]	4 (4)	259,78	6,39E+04	1,66E+04	4,27E-06	gi|121912479
Unknown	71,88	Unknown	hypothetical protein TVAG_073850 [Trichomonas vaginalis G3]	2 (2)	27,16	3,60E+05	5001,77	2,10E-06	gi|121904064
Unknown	38,77	Unknown	hypothetical protein TVAG_468600 [Trichomonas vaginalis G3]	2 (2)	28,26	6,93E+05	1,79E+04	6,75E-07	gi|121894474
Unknown	34,83	Unknown	hypothetical protein TVAG_437390 [Trichomonas vaginalis G3]	2 (2)	30,01	9,06E+05	2,60E+04	3,27E-06	gi|121916130
Unknown	33,62	Unknown	hypothetical protein TVAG_107130 [Trichomonas vaginalis G3]	2 (2)	39,66	1,78E+04	529,75	2,08E-05	gi|121891496
Unknown	30,06	Unknown	hypothetical protein TVAG_398490 [Trichomonas vaginalis G3]	3 (3)	47,12	5,19E+05	1,73E+04	9,12E-06	gi|121886424
Unknown	26,1	Unknown	hypothetical protein TVAG_237830 [Trichomonas vaginalis G3]	2 (2)	33,96	5,68E+04	2176,18	1,82E-05	gi|121916980
Unknown	25,28	Unknown	hypothetical protein TVAG_431160 [Trichomonas vaginalis G3]	2 (2)	36,23	2,02E+04	797,16	1,28E-06	gi|121896835
Unknown	23,96	Unknown	hypothetical protein TVAG_343940 [Trichomonas vaginalis G3]	2 (2)	32,59	1,42E+05	5940,33	2,39E-07	gi|121906390
Unknown	11,09	Unknown	hypothetical protein TVAG_474360 [Trichomonas vaginalis G3]	3 (3)	66,75	4,52E+04	4075,46	9,98E-07	gi|121899160
Unknown	10,63	Unknown	hypothetical protein TVAG_145680 [Trichomonas vaginalis G3]	2 (2)	31,61	8,98E+04	8450,01	2,49E-04	gi|121903522
Unknown	10,31	Unknown	hypothetical protein TVAG_050140 [Trichomonas vaginalis G3]	2 (2)	38,37	2,33E+04	2258,32	4,77E-06	gi|121902205

Unknown	10,08	Unknown	hypothetical protein TVAG_411320 [Trichomonas vaginalis G3]	2 (2)	43,82	1,38E+05	1,36E+04	3,38E-06	gi|121910013
Unknown	10,06	Unknown	hypothetical protein TVAG_346840 [Trichomonas vaginalis G3]	2 (2)	35,55	1,03E+05	1,03E+04	1,52E-06	gi|121892389
Unknown	9,83	Unknown	trichohyalin, putative [Trichomonas vaginalis G3]	2 (2)	34,76	2,48E+05	2,53E+04	4,74E-06	gi|121890270
Unknown	9,34	Unknown	unknown [Trichomonas vaginalis]	2 (2)	50,84	2,48E+04	2658,03	1,56E-04	gi|8886406
Unknown	8,69	Unknown	hypothetical protein TVAG_123890 [Trichomonas vaginalis G3]	5 (5)	91,09	2,15E+05	2,48E+04	2,21E-07	gi|121900942
Unknown	7,01	Unknown	hypothetical protein TVAG_324880 [Trichomonas vaginalis G3]	3 (3)	43,43	3,55E+05	5,07E+04	3,81E-06	gi|121896216
Unknown	6,9	Unknown	hypothetical protein TVAG_484970 [Trichomonas vaginalis G3]	2 (2)	35,41	3,85E+04	5571,69	3,74E-05	gi|121880058
Unknown	6,86	Unknown	hypothetical protein TVAG_080900 [Trichomonas vaginalis G3]	3 (3)	70,53	4,95E+05	7,22E+04	1,94E-04	gi|121900462
Unknown	6,86	Unknown	hypothetical protein TVAG_354770 [Trichomonas vaginalis G3]	2 (2)	33,78	1,51E+04	2207,47	4,52E-05	gi|121903451
Unknown	6,67	Unknown	hypothetical protein TVAG_092160 [Trichomonas vaginalis G3]	2 (2)	32,51	2,92E+05	4,37E+04	2,55E-06	gi|121885070
Unknown	6,36	Unknown	hypothetical protein TVAG_090740 [Trichomonas vaginalis G3]	4 (4)	69,63	3,51E+05	5,52E+04	3,33E-05	gi|121893329
Unknown	6,29	Unknown	hypothetical protein TVAG_034750 [Trichomonas vaginalis G3]	2 (2)	44,13	3,11E+04	4946,4	1,14E-06	gi|121889632
Unknown	5,77	Unknown	hypothetical protein TVAG_420130 [Trichomonas vaginalis G3]	2 (2)	35,41	9,36E+04	1,62E+04	3,57E-04	gi|121904444
Unknown	5,48	Unknown	hypothetical protein TVAG_454150 [Trichomonas vaginalis G3]	3 (3)	59,95	9,60E+04	1,75E+04	1,22E-05	gi|121912769
Unknown	5,06	Unknown	hypothetical protein TVAG_420220 [Trichomonas vaginalis G3]	2 (2)	32,07	2,24E+05	4,43E+04	9,75E-04	gi|121904453
Unknown	4,99	Unknown	hypothetical protein TVAG_249590 [Trichomonas vaginalis G3]	2 (2)	34,48	4,54E+04	9097,28	1,04E-04	gi|121917123
Unknown	4,72	Unknown	hypothetical protein TVAG_374940 [Trichomonas vaginalis G3]	3 (3)	83,67	2,95E+04	6247,27	2,64E-06	gi|121885719

Unknown	4,66	Unknown	hypothetical protein TVAG_425440 [Trichomonas vaginalis G3]	2 (2)	26,83	6,65E+04	1,43E+04	4,10E-06	gi|121882729
Unknown	4,6	Unknown	conserved hypothetical protein [Trichomonas vaginalis G3]	2 (2)	28,07	3,64E+04	7908,32	1,35E-06	gi|121903532
Unknown	4,41	Unknown	hypothetical protein TVAG_248380 [Trichomonas vaginalis G3]	2 (2)	36,55	1,79E+04	4056,04	4,11E-05	gi|121906564
Unknown	4,39	Unknown	conserved hypothetical protein [Trichomonas vaginalis G3]	2 (2)	36,44	5,76E+04	1,31E+04	9,43E-06	gi|121910686
Unknown	4,37	Unknown	hypothetical protein TVAG_103950 [Trichomonas vaginalis G3]	3 (3)	54,5	7,85E+04	1,79E+04	7,59E-07	gi|121888190
Unknown	3,95	Unknown	hypothetical protein TVAG_009150 [Trichomonas vaginalis G3]	2 (2)	28,39	8,08E+04	2,04E+04	1,32E-07	gi|121881711
Unknown	3,86	Unknown	hypothetical protein TVAG_391180 [Trichomonas vaginalis G3]	2 (2)	28,47	4,60E+04	1,19E+04	1,88E-05	gi|121915950
Unknown	3,83	Unknown	hypothetical protein TVAG_429790 [Trichomonas vaginalis G3]	2 (2)	77,15	1,63E+05	4,27E+04	4,88E-03	gi|121889097
Unknown	3,76	Unknown	hypothetical protein TVAG_231340 [Trichomonas vaginalis G3]	2 (2)	39,8	5,29E+04	1,41E+04	6,26E-05	gi|121895616
Unknown	3,69	Unknown	hypothetical protein TVAG_250870 [Trichomonas vaginalis G3]	2 (2)	36,5	3,37E+04	9124,25	4,60E-04	gi|121889502
Unknown	3,52	Unknown	hypothetical protein TVAG_497970 [Trichomonas vaginalis G3]	2 (2)	37,47	2,26E+05	6,43E+04	1,68E-05	gi|121885514
Unknown	3,5	Unknown	hypothetical protein TVAG_034380 [Trichomonas vaginalis G3]	3 (3)	51,15	1,64E+05	4,68E+04	1,72E-03	gi|121901242
Unknown	3,48	Unknown	hypothetical protein TVAG_132560 [Trichomonas vaginalis G3]	2 (2)	29,99	5,76E+05	1,65E+05	2,02E-03	gi|121886642
Unknown	3,47	Unknown	hypothetical protein TVAG_286280 [Trichomonas vaginalis G3]	2 (2)	70,56	3,36E+04	9673,65	2,13E-04	gi|121900413
Unknown	3,44	Unknown	hypothetical protein TVAG_440200 [Trichomonas vaginalis G3]	3 (3)	211,22	3,89E+05	1,13E+05	1,66E-03	gi|121891682
Unknown	3,4	Unknown	hypothetical protein TVAG_066230 [Trichomonas vaginalis G3]	2 (2)	29,36	2,55E+05	7,50E+04	4,71E-04	gi|121885580
Unknown	3,22	Unknown	hypothetical protein TVAG_222340 [Trichomonas vaginalis G3]	3 (3)	61,94	1,25E+05	3,87E+04	7,55E-05	gi|121883477

Unknown	3,16	Unknown	hypothetical protein TVAG_085630 [Trichomonas vaginalis G3]	2 (2)	38,97	1,30E+05	4,11E+04	9,40E-04	gi|121895549
Unknown	3,11	Unknown	hypothetical protein TVAG_295140 [Trichomonas vaginalis G3]	2 (2)	46,18	2,83E+04	9097,47	0,01	gi|121914059
Unknown	3,08	Unknown	hypothetical protein TVAG_013740 [Trichomonas vaginalis G3]	3 (3)	58,34	1,09E+05	3,54E+04	2,40E-03	gi|121916831
Unknown	3,05	Unknown	hypothetical protein TVAG_415360 [Trichomonas vaginalis G3]	2 (2)	34,89	5,79E+04	1,90E+04	1,44E-06	gi|121897998
