# Supplementary material for: Membrane associated proteins of two Trichomonas gallinae clones vary with the virulence
Source: PLoS One. 2019 Oct 24;14(10):e0224032. doi: 10.1371/journal.pone.0224032 (PMC6812828; doi:10.1371/journal.pone.0224032)
Supplement: S2 Table — Hypothetical proteins relatively quantified proteins from the enriched membrane proteins fraction for T. gallinae clonal cultures that are more abundant in clone R17-12 C1 with 2 or more identified peptides, ≥ 3 fold change and statistical significance (ANOVA, p-value < 0.01). P: number of identified peptides. (n): number of unique, non-conflicting peptides. Score: Total protein score (sum of individual peptides scores). (PDF) [file pone.0224032.s005.pdf]

Hypothetical proteins up-regulated in R17. Hypothetical proteins relatively quantified proteins from membrane and organelle membrane fraction for *T. gallinae* clonal cultures that are more abundant in clone R17-12 C1 with 2 or more identified peptides, ≥ 3 fold change and statistical significance (ANOVA, *p*-value < 0.01). P: number of identified peptides. (n): number of unique, non-conflicting peptides. Score: Total protein score (sum of individual peptides scores).

Predicted Function	Fold Change	Category	Description	P	Score	P178-13	R17-12	*p*-value	Accesion Number
Binding of large substrates	3,39	Cellular metabolism	hypothetical protein TVAG_269050 [Trichomonas vaginalis G3]	2 (2)	29,17	1,19E+04	4,01E+04	1,35E-03	gi|121903411
Binding of large substrates	3,23	Cellular metabolism	hypothetical protein TVAG_067470 [Trichomonas vaginalis G3]	2 (2)	27,16	5,57E+04	1,80E+05	5,48E-04	gi|121911914
Chromatin associated protein	8,37	Cellular component	hypothetical protein TVAG_176990 [Trichomonas vaginalis G3]	2 (2)	45,75	1,85E+04	1,55E+05	5,47E-07	gi|121893061
Cytoskeleton	15,03	Cellular component	conserved hypothetical protein [Trichomonas vaginalis G3]	2 (2)	72,72	2663,79	4,00E+04	7,90E-06	gi|121905200
Cytoskeleton	6,89	Cellular component	conserved hypothetical protein [Trichomonas vaginalis G3]	2 (2)	42,44	1,02E+04	7,06E+04	5,96E-05	gi|121905453
Cytoskeleton	3,8	Cellular component	hypothetical protein TVAG_166580 [Trichomonas vaginalis G3]	2 (2)	63,65	1277,02	4855,76	1,56E-05	gi|121916503
Energetic metabolism	3,98	Cellular metabolism	hypothetical protein TVAG_164890 [Trichomonas vaginalis G3]	17 (17)	1307,42	5,60E+05	2,23E+06	8,24E-07	gi|121886677
Hydrolase	10,34	Cellular metabolism	hypothetical protein TVAG_034780 [Trichomonas vaginalis G3]	2 (2)	34,23	1553,6	1,61E+04	7,15E-04	gi|121917683
Nucleotide binding	6,27	Cellular metabolism	hypothetical protein TVAG_069480 [Trichomonas vaginalis G3]	2 (2)	30,21	2,57E+04	1,61E+05	3,71E-05	gi|121901569
Nucleotide binding	3,3	Cellular metabolism	hypothetical protein TVAG_447350 [Trichomonas vaginalis G3]	2 (2)	43,77	1,69E+04	5,56E+04	6,27E-05	gi|121911949
Proteasome regulation	5,92	Cellular metabolism	hypothetical protein TVAG_105990 [Trichomonas vaginalis G3]	2 (2)	30,71	2130,53	1,26E+04	1,90E-03	gi|121882893
Proteasome regulation	5,61	Cellular metabolism	hypothetical protein TVAG_461850 [Trichomonas vaginalis G3]	2 (2)	39,8	865,64	4857,22	5,06E-03	gi|121890016
Protein binding	17,97	Cellular metabolism	hypothetical protein TVAG_201640 [Trichomonas vaginalis G3]	2 (2)	52,48	507,58	9119,92	4,14E-06	gi|121910322
Protein catabolism	11,64	Cellular metabolism	hypothetical protein TVAG_202850 [Trichomonas vaginalis G3]	2 (2)	38,18	1,32E+04	1,53E+05	2,75E-03	gi|121900799

Protein synthesis	3,69	Cellular metabolism	hypothetical protein TVAG_021170 [Trichomonas vaginalis G3]	11 (11)	185	4,37E+04	1,61E+05	1,35E-03	gi|121915383
Protein-protein interaction	5,76	Cellular metabolism	conserved hypothetical protein [Trichomonas vaginalis G3]	2 (2)	29,35	2,84E+04	1,63E+05	9,12E-03	gi|121880471
Protein-protein interaction	5,58	Cellular metabolism	hypothetical protein TVAG_295630 [Trichomonas vaginalis G3]	2 (2)	32,38	2193,21	1,22E+04	1,12E-03	gi|121895859
Protein-protein interaction	5,47	Cellular metabolism	hypothetical protein TVAG_272350 [Trichomonas vaginalis G3]	2 (2)	32,55	2393,81	1,31E+04	3,65E-03	gi|121891581
Protein-protein interaction	3,14	Cellular metabolism	hypothetical protein TVAG_072710 [Trichomonas vaginalis G3]	2 (2)	37,26	2,11E+04	6,62E+04	7,79E-04	gi|121884184
Ribosomal protein	4,87	Cellular component	hypothetical protein TVAG_183210 [Trichomonas vaginalis G3]	2 (2)	28,68	3040,94	1,48E+04	1,96E-04	gi|121918316
Translation	7,53	Translation	hypothetical protein TVAG_456340 [Trichomonas vaginalis G3]	2 (2)	31,98	1,58E+04	1,19E+05	6,78E-07	gi|121917224
Translation	6,9	Translation	hypothetical protein TVAG_369910 [Trichomonas vaginalis G3]	2 (2)	30,06	5705,69	3,93E+04	2,06E-05	gi|121897649
Unknown	Infinity	Unknown	hypothetical protein TVAG_178450 [Trichomonas vaginalis G3]	2 (2)	29,49	0	7837,09	1,72E-11	gi|121915008
Unknown	47,27	Unknown	hypothetical protein TVAG_172100 [Trichomonas vaginalis G3]	2 (2)	34,84	660,81	3,12E+04	2,90E-07	gi|121916171
Unknown	36,76	Unknown	hypothetical protein TVAG_281980 [Trichomonas vaginalis G3]	2 (2)	38,32	35,72	1313,32	5,59E-03	gi|121905674
Unknown	36,55	Unknown	hypothetical protein TVAG_191350 [Trichomonas vaginalis G3]	3 (3)	46,76	969,61	3,54E+04	1,95E-07	gi|121899991
Unknown	28,38	Unknown	hypothetical protein TVAG_306240 [Trichomonas vaginalis G3]	2 (2)	29,34	2,53E+04	7,19E+05	1,32E-08	gi|121913292
Unknown	15,37	Unknown	hypothetical protein TVAG_079530 [Trichomonas vaginalis G3]	2 (2)	31,49	3142,31	4,83E+04	2,97E-05	gi|121903733
Unknown	15,36	Unknown	hypothetical protein TVAG_274870 [Trichomonas vaginalis G3]	3 (3)	45,93	1,29E+04	1,99E+05	1,12E-04	gi|121905190
Unknown	14,89	Unknown	hypothetical protein TVAG_226060 [Trichomonas vaginalis G3]	2 (2)	32,47	2202,86	3,28E+04	4,29E-06	gi|121886529
Unknown	13,24	Unknown	hypothetical protein TVAG_198390 [Trichomonas vaginalis G3]	2 (2)	50,17	1,66E+04	2,19E+05	7,01E-07	gi|121916622
Unknown	9,82	Unknown	hypothetical protein TVAG_452000 [Trichomonas vaginalis G3]	2 (2)	30,83	1779,86	1,75E+04	4,25E-04	gi|121914488

Unknown	9,32	Unknown	hypothetical protein TVAG_311190 [Trichomonas vaginalis G3]	2 (2)	35,21	1147,38	1,07E+04	6,13E-05	gi|121885740
Unknown	9,21	Unknown	hypothetical protein TVAG_394820 [Trichomonas vaginalis G3]	5 (5)	88,08	6,42E+04	5,92E+05	2,13E-03	gi|121904361
Unknown	8,68	Unknown	hypothetical protein TVAG_094340 [Trichomonas vaginalis G3]	2 (2)	35,35	4652,59	4,04E+04	9,08E-06	gi|121917485
Unknown	8,54	Unknown	hypothetical protein TVAG_223640 [Trichomonas vaginalis G3]	4 (4)	77,95	1,34E+05	1,14E+06	2,31E-04	gi|121902309
Unknown	7,07	Unknown	hypothetical protein TVAG_475480 [Trichomonas vaginalis G3]	3 (3)	48,62	8793,86	6,22E+04	1,79E-06	gi|121917849
Unknown	6,7	Unknown	hypothetical protein TVAG_097910 [Trichomonas vaginalis G3]	5 (5)	90,12	1,91E+04	1,28E+05	3,11E-04	gi|121908343
Unknown	6,15	Unknown	hypothetical protein TVAG_165240 [Trichomonas vaginalis G3]	2 (2)	54,28	9903,05	6,09E+04	1,54E-04	gi|121911058
Unknown	6,1	Unknown	hypothetical protein TVAG_278990 [Trichomonas vaginalis G3]	2 (2)	27,14	6225,32	3,80E+04	7,04E-04	gi|121880711
Unknown	5,71	Unknown	hypothetical protein TVAG_339270, partial [Trichomonas vaginalis G3]	2 (2)	66,29	1,21E+04	6,90E+04	5,19E-06	gi|121893354
Unknown	5,51	Unknown	hypothetical protein TVAG_211540 [Trichomonas vaginalis G3]	2 (2)	28,63	2,88E+04	1,59E+05	1,02E-05	gi|121901696
Unknown	5,34	Unknown	hypothetical protein TVAG_453100 [Trichomonas vaginalis G3]	2 (2)	31,75	2,24E+04	1,19E+05	3,70E-07	gi|121899431
Unknown	5,03	Unknown	hypothetical protein TVAG_258250 [Trichomonas vaginalis G3]	2 (2)	28,53	9797,97	4,93E+04	4,65E-06	gi|121905903
Unknown	5,02	Unknown	hypothetical protein TVAG_014360 [Trichomonas vaginalis G3]	2 (2)	41,88	4538,25	2,28E+04	5,52E-04	gi|121916891
Unknown	4,56	Unknown	hypothetical protein TVAG_450670 [Trichomonas vaginalis G3]	2 (2)	29,81	6571,51	2,99E+04	1,22E-05	gi|121898402
Unknown	4,37	Unknown	hypothetical protein TVAG_053070 [Trichomonas vaginalis G3]	2 (2)	37,07	6,74E+04	2,95E+05	9,74E-05	gi|121894239
Unknown	4,29	Unknown	hypothetical protein TVAG_234990 [Trichomonas vaginalis G3]	2 (2)	28,42	1,47E+04	6,28E+04	5,00E-07	gi|121912801
Unknown	4,2	Unknown	conserved hypothetical protein [Trichomonas vaginalis G3]	2 (2)	27,32	5,01E+04	2,10E+05	1,70E-05	gi|121882494
Unknown	4,15	Unknown	hypothetical protein TVAG_182030 [Trichomonas vaginalis G3]	2 (2)	31,56	5606,64	2,33E+04	3,68E-08	gi|121894117

Unknown	4,04	Unknown	hypothetical protein TVAG_410430 [Trichomonas vaginalis G3]	2 (2)	38,16	1,17E+04	4,74E+04	2,48E-07	gi|121906124
Unknown	4,01	Unknown	hypothetical protein TVAG_042770 [Trichomonas vaginalis G3]	2 (2)	27,67	4,14E+04	1,66E+05	8,24E-04	gi|121887453
Unknown	4	Unknown	hypothetical protein TVAG_101120 [*Trichomonas vaginalis* G3]	2 (2)	39,06	1,80E+04	7,21E+04	5,98E-08	gi|121914597
Unknown	3,92	Unknown	hypothetical protein TVAG_124350 [Trichomonas vaginalis G3]	2 (2)	38,81	8475,14	3,32E+04	2,03E-04	gi|121884217
Unknown	3,66	Unknown	hypothetical protein TVAG_083270 [Trichomonas vaginalis G3]	2 (2)	39,28	1,45E+04	5,32E+04	7,45E-05	gi|121913667
Unknown	3,6	Unknown	hypothetical protein TVAG_108950 [Trichomonas vaginalis G3]	3 (3)	62,25	4,53E+04	1,63E+05	2,32E-05	gi|121896591
Unknown	3,51	Unknown	hypothetical protein TVAG_252720 [Trichomonas vaginalis G3]	2 (2)	29,49	1,12E+04	3,93E+04	2,44E-04	gi|121910614
Unknown	3,47	Unknown	hypothetical protein TVAG_031830 [Trichomonas vaginalis G3]	3 (2)	54,04	1,53E+05	5,31E+05	4,95E-07	gi|121898598
Unknown	3,45	Unknown	hypothetical protein TVAG_181960 [Trichomonas vaginalis G3]	2 (2)	30,5	4116,95	1,42E+04	3,19E-05	gi|121894110
Unknown	3,34	Unknown	hypothetical protein TVAG_472390 [Trichomonas vaginalis G3]	3 (3)	59,72	8958,55	2,99E+04	2,42E-06	gi|121894522
Unknown	3,33	Unknown	hypothetical protein TVAG_098730 [Trichomonas vaginalis G3]	2 (2)	45,64	6053,97	2,01E+04	1,86E-04	gi|121901143
Unknown	3,23	Unknown	hypothetical protein TVAG_476440 [Trichomonas vaginalis G3]	2 (2)	33,54	1,00E+04	3,24E+04	1,88E-03	gi|121917944
